# Characterization and validation of EHR computable phenotypes for Long COVID using patient-reported symptoms: insights from the nationwide RECOVER program

**DOI:** 10.1093/jamia/ocag115

**Published:** 2026-07-24

**Authors:** Victor M Castro, Vivian Gainer, Nich Wattanasin, Andrew Cagan, Ana Holzbach, James Chan, Leora Horwitz, Rachel Kenney, Ivan Diaz, Hannah Mandel, Shannon Wuller, Mady Hornig, Lisa O’Brien, Andrew Wylam, James Doster, Richard A Moffitt, Emily Pfaff, Mark G Weiner, Sajjad Abedian, Michael Koropsak, Sairam Parthasarathy, Hanieh Razzaghi, Justin Manjourides, Elizabeth W Karlson, Shawn N Murphy, of the RECOVER Consortium, Lisa T Newman, Julie Abella, Quinn Barnette, Christine Bevc, Jennifer Beverly, Patricia Ceger, Julie Croxford, Emily Cunningham, Mike Enger, Katie Fain, Tonya Farris, Sean Hanlon, David Hines, Vicki Johnson-Lawrence, Kevin Jordan, Craig Lefebvre, Beth Linas, Bryan Luukinen, Meisha Mandal, Nikki J McKoy, Susan Nance, Ashleigh Oakland, Demian Pasquarelli, Claire Quiner, Rita Sembajwe, Gwendolyn Shaw, Vanessa Thornburg, Kendall Tosco, Hannah Wright, Rachel S Gross, Judith S Hochman, Leora I Horwitz, Stuart D Katz, Andrea B Troxel, Lenard Adler, Precious Akinbo, Ramona Almenana, Ola Bello, Sultana Bhuiyan, Nina Blachman, Ryan Branski, Jasmine Briscoe, Shari B Brosnahan, Elliott Bueler, Yvette Burgos, Nina Caplin, Domonique N Chaplin, Yu Chen, Shen Cheng, Peter Choe, Jess Choi, Alicia Chung, Richard Church, Stanley Cobos, Nakia Croft, Angelique Cruz Irving, Phoebe Del Boccio, Iván Díaz, Vishal Doshi, Samantha Ebel, Arline Faustin, Elias Febres, Jeffrey Fine, Sandra Fink, Jennifer Frontera, Richard Gallagher, Alejandra Gonzalez-Duarte, Denise Hasson, Sophia Hill, Shahidul Islam, Stephen Johnson, Neha Kansal, Rachel Kenney, Deepshikha Kewlani, Michelle F Lamendola-Essel, Gregory Laynor, Terry Leon, Zoe A Lewczak, Janelle Linton, Max Logan, Nadia Malik, Lia Mamistvalova, Hannah Mandel, Gabrielle Maranga, Patenne D Mathews, Aprajita Mattoo, Tony Mei, Alan Mendelsohn, Emmanuelle Mercier, Patricio Millar Vernetti, Marc Miller, Maika Mitchell, Andre Moreira, Praveen C Mudumbi, Erica Nahin, Nandini Nair, Joseph Nekulak, Kellie Owens, Brendan Parent, Nandan Patibandla, Peter Petrov, Radu Postelnicu, Isabelle Randall, Priyatha Rao, Amy Rapkiewicz, JohnRoss Rizzo, Johana Rosas, Chelsea Rose, Christina Saint-Jean, Michelle Santacatterina, Binita Shah, Aasma Shaukat, Naomi Simon, Aylin Simsir, Miranda Stinson, Wenfei Tang, Vasishta Tatapudi, Sujata Thawani, Mary Thomas, Lorna Thorpe, MeeLee Tom, Ethan Treiha, Jennifer Truong, Mmekom Udosen, Jessica Velazquez-Perez, Patricio M Vernetti, Crystal Vidal, Anand Viswanathan, Crystal Wong, Marion J Wood, Shonna H Yin, Chloe Young, Jonah Zaretsky, Susanna Zavlunova, Andrea S Foulkes, Elizabeth W Karlson, Shawn Murphy, Shreya Ahirwar, Layne L Ainsworth, Rachel Atchley-Challenner, Paul Avilach, Trisha T Balan, Nicholas Benik, Barbara Benoit, Marie-Abèle C Bind, William J Bonaventura, Natalie Boutin, Beverly Brion, Andrew Cagan, Tianrun Cai, Victor M Castro, Xander R Cerretani, James G Chan, David Cheng, Lori B Chibnik, Mark Ciriello, Karen Costenbader, Dimitar S Dimitrov, Hossein Estiri, Meng Fang, Maria E Fayad, Candace H Feldman, Vivian Gainer, Bhaswati Ghosh, Randy Gollub, Zoe Guan, Alan Harris, Karl Helmer, Andrew Hendrix, Ana Holzbach, Weixing Huang, Daniel Kaufman, Diane Keogh, James D Kerr, Jeffrey G Klann, Aparna Krishnamoorthy, Jessica A Lasky-Su, Katherine P Liao, Jessica Lin, Doug MacFadden, Anupama Maram, Megan W Martel, Michael Mendis, Reeta Metta, Jonathan Monteiro, Eduardo Morales, Richard E Morse, Marc-Danie Nazaire, Gregory Neils, James Norman, Henry H Paik, Deepti Pant, Heekyong Park, Zihan Qian, Harrison T Reeder, Kathleen Rossi-Roh, Leah M Santacroce, Daniel Schwartz, Caitlin A Selvaggi, William Simons, Lynn A Simpson, Jacob Sitak, Zachary Strasser, Ayesha Tariq, Tanayott Thaweethai, Madeleine Thorn, Philip Trewett, Dustin Van Fleet, Kavishwar B Wagholikar, Haoyang Wang, Taowei D Wang, Nich Wattanasin, Griffin Weber, Michael A Williams, Mine Cicek, Thomas J Flotte, Erik M Boysen, Nancy Chang, Evan Ellingworth, Erika Frisch, Teagan Iwanski, Karla Kopp, Gary Welch, Abdullahi Yusuf, Nicole Zahnle, Marta Cerda, Victor H Clash, Felicia Davis Blakley, Brittany Taylor, Mike Zissis, Teresa Akintonwa, Frank Blancero, Heather-Elizabeth Brown, Megan Carmilani, Debra Copeland, Yvonka Hall, Kevin Kondo, Lydia Lerma, Jacqui Lindsay, Heather Marti, Christine Maughan, Tony Minor, Hyatt Vincent, Jeffrey P Burns, Serena Spudich, Charles Bailey, Mine Cicek, Melissa M Cortez, Felicia Davis Blakley, Andrea S Foulkes, David Goff, Stuart D Katz, Jessica Lasky-Su, Torri D Metz, Lisa T Newman, Igho Ofotokun, Sudha Seshadri, Melissa Stockwell, James Stone, Brittany D Taylor, P J Utz, Neely A Williams, Andra L Blomkalns, Valerie J Flaherman, Ingrid V Bassett, Rebecca G Clifton, Hannah Davis, Nathan Erdmann, Margot Gage Witvliet, Mark P Goldberg, Zoe Guan, Mamta K Jain, Jonathan D Klein, Gregory Laynor, Thomas Martinez, Rebecca McGrath, Sairam Parthasarathy, Priscilla Pemu, Jacqueline Rutter, David Warburton, Hassan Ashktorab, Christine Bevc, Karyn Bishof, Yu Chen, Lori B Chibnik, Dani Dumitriu, Jennifer Frontera, Paul Goepfert, Sylvie Goldman, Stephen Hewitt, Matt Huentelman, Barbara Karp, Jerry A Krishnan, Marrah Lachowicz-Scroggins, Bruce D Levy, Miriam Merad, Shawn Murphy, Janko Nikolich-Zugich, Laura A Pace, Alice Perlowski, Brian Reeves, Juan Salazar, Sujata Thawani, Hannah Valantine, Drenna Waldrop, Jenny E Han, Vincent C Marconi, Ighovwerha Ofotokun, Rachel E Patzer, Tiffany A Walker, Rachael Abraham, Franchesca A Aguilar, Ghazal Ahmadi-Izad, Dilshad R Ahmed, Alicarmen Alvarez, Blake Anderson, Walter D Asencios, Casey L Beaty, Brahmchetna Bedi, Jasmine A Berry, Donchel Boone, Mary Bower, James D Bremner, Corbin Brent, Ke’Ara Brown-Smith, Rachel Bull, Gustavo Capo, Kelechi Carl-Igwe, Calista Chitadze, Nachi Chukwumerije, Erna Clyburn, Shelby Collins, Julie Costello, Grace Couture, Angel Craft, Xiangqin Cui, Carlos del Rio, Joshua F Detelich, Cartia Dixon, Jeanne Dow, D’Andrea Doyle, Jannah Elchommali, Imani Elsey, Nicole Franks, Julia Gallini, Evan Gutter, Jess Harding, Liliana Hernandez, Carla Holloway, Cynthia Ifejika, Rijalda Jasarevic, Vidhi N Javia, Mykayla Jeter, Yasha Joseph, Monica Juarez, Caitlin M Kirkpatrick, Athena Koumanelis, Shilpa Krishnan, Jose D Leon, Valerie Lew, Cheryl L Maier, Nour Makkaoui, Mara Maroney, Christopher F Martin, Loice Mbogo, Atuarra McCaslin, Jerrod McIntyre, Abeer Moanna, Miranda Montoya, Elena Morales, Caitlin A Moran, Calista Murray, Roslin Nelson, Tran Nguyen, Bukkie Ojoawo, Eileen Osinski, Sofia Oviedo, Yolanda Paredes-Gaitan, Michael Prude, Grace Ramakrishnan, Paulina A Rebolledo, Marjorie Roberts, Keysha Robinson, Chantrice Rogers, Nadine G Rouphael, Charles Searles, Marni Segall, Anand Shah, Ruvina Silva, Cheryl Simpson, Krystal Simpson-Derrell, Talib Sirajud-Deen, Jacob Stroud, Mehul S Suthar, Cory Sylber, Ashley Sylvera, Larissa J Teunis, Kodasha M Thomas, Kehmia Titanji, Christopher Toy, Alex Truong, Viola Vaccarino, Kris Varney, Kartavya Vyas, Kurt Vyas, Max Walkow, Tamara Wesley, Juton R Winston, Terra J Winter, Cherry Wongtrakool, Sushma K Cribbs, Olivia Collins, Christina N Fournier, Anyssa G Francis, Tina P Hang, Ketteria D Ingram, Jordi Lainez, Leon Rubinsztain, Chengcheng (Cindy) Ye, Howa Yeung, Zanthia Wiley, Arijan Ager, Natalie Gray, Ash Grimes, Daniel Gromer, Lauren N Hewitt, Christopher Huerta, Nadiya Jiwa, Brandi Johnson, Colleen Kelley, Lana Khalil, Dean Kleinhenz, Alexandra Koumanelis, Rebecca Kozoman, Matthew A Lee, Kennedy C Lewis, Matthew Litvack, Tsungirirai Maramba, Christina Mehta, Bernadine Panganiban, Kazi Rahman, Veronica E Smith, Andre Stringer, Maliya Tolbert, Jessica Traenkner, Kristen Unterberger, Heqiong Wang, Erika Wimberly, Qian Yang, A Blythe Ryerson, Zenisha Bain, Rebecca Hjortsberg, Alex F Hudgins, Monica Martinez, Robert B Neuman, Lauren Nisotel, Sierra G Thompson, Chiagoziem Agu, Priscilla Pemu, Amir Afani, Concilia Ariri, Annette Dandy, Kaysha Harper, Carmel Ibeawuchi, Brianna Lawrence, Jan Morgan-Billingslea, Elizabeth I Ojemakinde, Karina Smith, Bruce D Levy, Masanori Aikawa, Lindsey Baden, Gaston Baslet, Lindsey Bennett, Shamik Bhattacharyya, Julie Buring, Rebecca E Cagnina, Li Qing Chen, Cheryl R Clark, Pieter Cohen, Charles Czeisler, Peter Estill, Elizabeth Gay, Jessica Hong, Daniela Lamas, Sarina Lay, Nomi Levy-Carrick, JoAnn Manson, Tobasom Monafrared, Carrie Pistenmaa, Susan Redline, Elijah J Remis, Daniel Schilkrut, Howard D Sesso, Scott Solomon, Jeffrey A Sparks, Lia L Spencer, David Systrom, Phyo Phyo Min Thu, David Walt, George Washko, Maureen Whittelsey, Rebecca Wiener, Ingrid V Bassett, George A Alba, Taing N Aung, Kathleen Fitch, Leo C Ginns, Jennifer Haas, Yanxiang Hu, Boris D Juelg, Diane G Kanjilal, Arthur Y Kim, Elizabeth B Klerman, Gregory Lewis, Awo Musa, Bisola Ojikutu, Roy Perlis, Jonathan Rosand, Zachary S Wallace, Dean Xerras, Danielle Zionts, Janet M Mullington, Ai-Ris Collier, Tamara Fong, Monika Haack, Kristine S Hauser, Jason H Maley, Yuri Quintana, Lynn Shaughnessy, Kathryn Stephenson, Robert J Thomas, Robert Torres, Jai G Marathe, Elizabeth Duffy, Naomi Hamburg, George T O’Connor, Haihua Zhang, Janice John, Amberly Ticotsky, Honorine D Ward, Deborah Blazey-Martin, Maher Ghamloush, Michael Jordan, Laura Kogelman, Terry Marryshow, Nathaniel Erdmann, Emily B Levitan, Alan T Tita, Donna Armstrong, Susan E Binkley, Kenneth Blackwell, Annalia Causey, Felice Cook, Julio Domingo, Conner Donahue, Maitlyn Eady, Jeffrey Edberg, Kentevious Forehand, Patrick Frazier, Noah Garcia-McClaney, Melissa Garner, Brandon Gray, Wanda Hall, Cady Hart, Camden L Hebson, Bertha Hidalgo, Brian Higgins, Kaylen Holtzapfel, Gina Horton, Alexis Jinright, Suzanne E Judd, Teri Kennedy, Leigh Kirkwood, Megan Maier, Patricia McCormack, Kevin Mitchell, Aoyjai Montgomery, Dorothy Nieters, Myriam Peralta-Carcelen, Juan P Pilco, Leigh Powell, Jacob Royster, Rachael Shevin, Sidney Skipworth, Leah Spurgeon, Courtney Steele, Jane Vines, Gregory Ware, Rosanne Wilson, Dana Woodruff, Brandon Young, Mark Gillespie, Casey L Daniel, Jamie Hansel, Jing Wu, Thomas W Carton, Lucio Miele, Todd Brown, Erica Sutherland, Jyotsna Fuloria, Paula Datri, Michael Hagensee, Cathryn Leggio, Allen Perkins, Amber Trauth, Siobhan Trotter, Alexander Van Deerlin, Sharon Weiser, Madeline Young, Hassan Ashktorab, Hassan Brim, Adeyinka O Laiyemo, Zaki A Sherif, Saima Durrani, Ali Nezamloo, Julius Ngwa, Noelle Njoku, Monique P Gentil, Alem Mehari, Akbar Solemani, Linda Chang, Paul Thuluvath, Mhret Alemu, Jordan Anderson, Mahak Chauhan, Sung Cho, Karli Goodman, Gandi Lanke, Ralph Lebron, Anurag Maheshwari, Jina Ok, Chau To, Sally L Hodder, James M Bardes, Daphne Dominique-Villanueva, Joy Juskowich, Rebecca Reece, Arif Sarwari, Judd Shellito, Michael Hagensee, Lucio Miele, Frank L Greenway, John P Kirwan, Gabrielle Rodemann, Clifford J Rosen, Abigail Arruda, Ivette F Emery, Theresa Roelke, Paul Berger, Lora Black, Susan E Hoover, Brian Tjarks, Vivian Fonseca, Shaveeta Gupta, Michele Longo, Mei Yang, Cecilia M Shikuma, Dominic C Chow, Louis MarGangcuangco, Mario Castro, Charles Bengtson, Theresa Howard, Brandon Koontz, Leslie A Spikes, Christopher Simmons, Sidney W Whiteheart, Beth Garvy, Jeremy P Wood, Gailen D Marshall, Vishnu Garla, Joy Kuebler, Utsav Nandi, Andrew Vasey, David E Warren, John D Dickinson, Timothy M VanWagoner, Amanda Bogie, Daniel J Heyanka, Judith A James, James Scott, Fatima I Sukhera, Carlos A Luciano Roman, Carmen Irizarry, Sigrid Perez Frontera, Jorge Santana Bagur, Tina Vazquez, Jonathan D Klein, Jerry A Krishnan, Janet Y Lin, Naoko Muramatsu, Bellur S Prabhakar, Heather M Prendergast, Terry L Vanden Hoek, Dara Adams, Aileen Baker, Sunni Barbera, Sanjib Basu, Susan Bleasdale, Andrew D Boyd, Taylor Breiter, Irina A Buhimschi, Michael D Carrithers, Rashmika Chalamalla, David Chestek, Judith A Cook, Dawood Darbar, Raktima Dasgupta, Felicia Davis Blakley, Julie A DeLisa, Kathleen R Diviak, Meghan F Donlon, Mark S Dworkin, Angela Ellison, Clarie Flanigan, Michael B Freedman, Lynn B Gerald, Wayne H Giles, Howard S Gordon, Bayan Hammad, Sharon Hasek, Wendy Hasse, Martyna Hryniewicka, Sai D Illendula, Nahed Ismail, Akash Jain, Kyle J Jennette, Grace Kadubek, Denise Kent, Denise A Kent, Keri S Kim, Pavitra Kotini-Shah, Lucia Large, James Lash, Jun Lu, Abeer M Mahamed, Sergey Malchenko, Miriam Martinez, Cammeo Mauntel-Medici, Mark McCauley, Martha Menchaca, Robin Mermelstein, David J Moreno, Liam Morrissy, Hugh Musick, Lourdes Norwick, Richard M Novak, Marilyn Ortiz, Khushboo Patel, Nicolas L Perez, Neil H Pliskin, Sam Pope, Bharati Prasad, Barbara Predki, John G Quigley, Ramaswamy Ramchandran, Ana Ramirez, Sarah Rappe, Jalees Rehman, Matthew Rowley, Gowrisree Rudraraju, Melissa Rutherfoord, Jennifer A Sculley, Jerisha Smith-Mack, Jun Sun, Nancy Tartt, Laura Villanueva, Sara Warfield Kelly, Cemal Yazici, Marta Certa, Erica Chessier, Emily Everett, Elijah Kindred, Pastor C Harris, Praveen Sudhindra, Lela Olds, Lisa Aponte-Soto, Marina Del Rios, Maya Z Diaz, Alejandra L Ibanez, Cesar Rolon, Savannah Cranford, Daniel Brown, Jennifer Dixon, Lisa Gale, Savannah Hammerl, Kimberly Hartwig, Abhigna Madineni, Peyton Swearingen, Monica Hendrickson, Seth Noland, Tracy Terlinde, Brianna Hobbs, Sarah A Stewart de Ramirez, Dawn Bolliger, Jerusha Boyineni, Praneeth Chebrolu, Hannah L Curry, Sarah E Donohue, Sherrie Edmonds, Sara W Kelly, Phoebe Maholovich, Samer B Sader, Tiffany Thompson, Hannah Welter, Brittany Woolley, John Hafner, Keith A Hanson, Robert Hutton, Alexander W Charney, Patricia Kovatch, Miriam Merad, Girish N Nadkarni, Juan P Wisnivesky, Judith A Aberg, Steven Ascolillo, Emilia Bagiella, Logan Bartram, Jacqueline Becker, Noam D Beckmann, Ashley Bendl, Benjamin K Chen, Alyssa Civil, Ginger Y Crawford, Kaberi Dhar, Lorraine Evo-Ortega, Daniel Fierer, Emily J Gallagher, Adolfo Garcia-Sastre, Sacha Gnjatic, Ian Gray, Sabina Guliyeva, Lori Harvey-Ingram, Julia Herrera-Moreno, Matthew Hill, Carol R Horowitz, Rachel Jackson, Din Kastrat, Anu Lala-Trindade, Jenny Lin, Kathryn Marcon, Dara Meyer, Janice Morinigo, Benjamin H Natelson, Maya Nussenzweig, Tiffani Padua, David Putrino, Lynne Richardson, Scott Russo, Alan C Seifert, Abdullah Serri, Jordan Walker, Michell Yee, Lucinda Bateman, Rachel Hess, Dongngan T Truong, Natalya Alekhina, Jackson Barlocker, Jeanette P Brown, Melissa Cortez, Dagny K Donohue, Julio C Facelli, Ramkiran Gouripeddi, Jessica A Hermansen, Jace D Johnny, Ashton M Lindsay, Jennifer Lloyd, Yue Lu, Juliemar C Medina, Sarah Shizuko Morimoto, Laura A Pace, Danielle M Rius, Mary Beth Scholand, Kevin S Shah, Nasser Sharareh, Adam M Spivak, Caitlyn Stringham, Joel D Trinity, Matt Velinder, Lisa J Weaver, Josh N West, Lucinda Bateman, Suzanne D Vernon, Sara J Deakyne Davies, Edward M Gardner, Kaitlin E Buck, Erick Gonzalez Rivas, Kellie L Hawkins, Judy L Oakes, Wvalter Sanchez Vazquez, Benjamin D Horne, Kirk Knowlton, Scott C Woller, Bailee Aguirre, Jeff Anderson, Tami Bair, Lindsay Bosh, Lorlie Evans, Chase Garrett, Dixie Harris, Katherine Herrera, Leslie Iverson, McKenna M Jensen, James Juan, Stacey Knight, Lindsay Leither, Heather Maestas, Heidi T May, Shyanne Zubal, Kristine M Erlandson, Ron J Sokol, Marisa Brightman, Debra Davis, Elen M Feuerriegel, Harrison Z Fudge, Lohit Garg, Janine Higgins, Sarah E Jolley, Tim Lockie, Sean A McCandless, Chloe Pitch, Jane E Reusch, Brook Thurman, Huong Tran, Shelby C West, Naomi P Friedman, Katelyn R Ludwig, Lauren A Decker, Hengameh Raissy, Natalie L Adolphi, David A Archuleta, Steven B Bradfute, Rebecca Brito, Jamie Elifritz, Noella D Garcia-Soberanez, Frederick D Gentry, Michelle S Harkins, Noah I Martinez, Lorenzo A Montoya, Alisha N Parada, Davin K Quinn, Alfredo Ramos, Elyce B Sheehan, Irena S Treacher, Grace A McComsey, Cara Adams, John Andrefsky, Ornina Atieh, Jhony Baissary, Nicholas Boldt, Kamal Chemali, Emily Ciborek, Ann Conrad, Brian D’ Anza, Joviane Daher, Kathryn DiFrancesco, Theresa Foster (Rodgers), Michelle Gallagher, Amit Gupta, Jami Harrill, Paul Harris, Frank Jacono, Jordyn Kelly, Ziad Koberssy, Rohini Kumar, Danielle Labbato, Joaquin Lim, Kimberly Pettinato, Michael Rodgers, Breandan Rosolia, Arnab Roy, Sravan Sattiraju, Sarah Scott, Beth Smith, Viral Tejani, Megan Tribout, George Yendewa, Nora G Singer, Mirna Ayache, Emma Barnboym, Alexis Brown, Hailey Chesnick, Marissa Edminston, Carla Greenwood, Maricela Haghiac, Elizabeth Kaufman, Ketrin Lengu, Rebecca Lowenthal, Shahdi Malakooti, Christine Oleson, Ann Pearman, Allison Rizea, Elisheva Weinberger, James R Heath, Conor Brennan, Rick Edmark, Vanessa Gutierrez, Jennifer Hadlock, Sarah Li, Andrew T Magis, Connor McDonald, Kim M Murray, Lee Rowen, Dan Yuan, Peter Chen, Aghigh Banitaba, Antonina Caudill, Tananshi Chopra, Lea Dahlke, Mario Diaz, Lasya Gudipudi, Susan Jackman, Brittany Mattison, Matthew Modes, Chloe Nelson, Chidimma Nwafor, Tanyalak Parimon, Ethan Pascual, Nancy Salinas, Sam Torbati, Sara Watson, Katherine R Tuttle, Radica Alicic, Joni Baxter, Sarah Emerson, Susan Hood, Kelli Kuykendall, Shane White, Lauren E Wilcox, Jason D Goldman, Heather A Algren, James Del Alcazar, Alexandria M Duven, John Kaneko, Paula Manner, Ashley Okada, Rachel Poussier, Richard Satira, Julie A Wallick, Helen Y Chu, Anna Elias-Warren, Alex Harteloo, Jennifer K Logue, Kathryn McCaffrey, Helen Nguyen, Anoop M Nambiar, Thomas F Patterson, Jennifer S Potter, Marzieh Salehi, Kumar Sharma, Monica Verduzco-Gutierrez, Reed Anderson, Azaneth Arellanes, Rose A Barajas, Suneet P Chauhan, Geoffrey D Clarke, Cheryl E Farner, Melinda S Fischer, Mark P Goldberg, Gabrielyd Hastings, Patricia Heard, Jessica Hernandez, Italia Herrera, Edgar Infante, Hillary Johnson, Johnnie Jones, Dean L Kellogg, Ellen Kraig, Lisa Longoria, Emeka Okafor, Jan E Patterson, Alexis Pinones, W B Reeves, Irma Scholler, Sudha Seshadri, Pankil Shah, Dimpy P Shah, Marlaysha Smith, Bridgette Soileau, Pamela Solis, Carmen Stoebner, Michael Sullivan, Barbara S Taylor, Robin Tragus, Joel Tsevat, Keren Hasbani, George R Saade, Jordan Barnaba, Gustavo S Hernandez Martinez, Cecilia Recabarren, Kavita Sharma, Claudia C Paredes, Stephanie Alvarado, Andre Kumar, Yvonne Maldonado, Paul J Utz, Catherine A Blish, Minjoung Go, Prasanna Jagannathan, Upinder Singh, Neera Ahuja, Andra L Blomkalns, Hector Bonilla, Richard Brotherton, Kimberly Clinton, Vaidehi Dingankar, Linda N Geng, Francois Haddad, Christopher Jamero, Kathryn Jee, Xiaolin K Jia, Naresh Khurana, Mitchell G Miglis, Ellen O’Conor, Kelly Olszewski, Divya Pathak, Orlando Quintero, Corey Saperia, Jake Scott, Alfredo E Urdaneta, Mary R Varkey, Janko Z Nikolich, Sairam Parthasarathy, Kacey C Ernst, Denise R Esquivel, David T Harris, Stefanie Harris, Michael Hernandez, Harvey Hsu, Michelle James, Maria Karnafel, Kenneth S Knox, Alison Koleski, Bonnie LaFleur, Brenda Lambert, Sicily LaRue, Karen Lutrick, Nirav Merchant, Christopher Morton, Jarrod M Mosier, Toluwanimi Olorunnisola, Jeanette Peralta, William ( Pilling, Kristen Pogreba-Brown, Franz P Rischard, Lee T Ryan, Terry Smith, Manuel Snyder, Vignesh Subbian, Kyle Suhr, Eric M Reiman, Joyce K Lee-Iannotti, Lynn Autry, Hassan Beydoun, Sabine Borwege, Jacquelynn Copeland, Marjorie DiLise-Russo, Susan Fadden, Isaias Gomez, Garrett Grischo, William Hartley, Leah Hillier, Hira Ismail, Stephanie Iusim, Michelle James, Mrinalini Kala, Daniel Kim, Ganesh Murthy, Samuel Unzek, Sheila Vadovicky, Sharry Veres, Christian Bime, Julie Cremer, Lillian Hansen, Trina Hughes, Saher Khalid, David Lieberman, Francisco Soto, Cathleen Wilson, Alyssa Zapien, Steven G Deeks, John D Kelly, Jeffrey N Martin, Michael J Peluso, Grace Anderson, Khamal Anglin, Urania Argueta, Kofi Asare, Melissa Buitrago, Celina Chang Song, Alexus Clark, Emily Conway, Nicole Del Castillo, Monika Deswal, Matthew S Durstenfeld, Elnaz Eilkhani, Avery Eun, Emily Fehrman, Tony Figueroa, Diana Flores, Halle Grebe, Timothy J Henrich, Rebecca Hoh, Priscilla Hsue, Beatrice Huang, Rania Ibrahim, Marian Kerbleski, Raushun Kirtikar, Megan T Lew, James Lombardo, Monica Lopez, Michael Luna, Carina Marquez, Sadie Munter, Lynn Ngo, Jesus Pineda-Ramirez, Aric Prather, Kim Rhoads, Antonio Rodriguez, Justin Romero, Dylan Ryder, Matthew So, Ma Somsouk, Viva Tai, Brandon Tran, Julian Uy, Daisy Valdivieso, Deepshika Verma, Meghann Williams, Andhy Zamora, Andrea B Troxel, Phoebe Del Boccio, Michelle F Lamendola-Essel, Chloe Young, James R Stone, Faye Victoria C Casimero, Vanessa C Hannay, Aram J Krauson, Iris A Lopez, Maria Martinez-Lage Alvarez, Robert F Padera, Mine Cicek, Thomas J Flotte, Erik M Boysen, Nancy Chang, Evan Ellingworth, Erika Frisch, Teagan Iwanski, Karla Kopp, Gary Welch, Abdullahi Yusuf, Nicole Zahnle, Kristen Thomas, Arline Faustin, Timothy Shepherd, Jonathan Melamed, Kevin Fitzpatrick, Moira Paskett, Marena Soliman, Carolyn Glass, Ameera Afifi, Beth Handel, Paul V Benson, Ayat Abdelsalam, Silvio Litovsky, Darin Trelka, Dezhi Wang, Avi Rosenberg, Katie Flickinger, Jowaly Schneider, Daniel Gallego Umana, Natalie Adolphi, Stacy Catanach, Hadya Khawaja, Kyla Sorensen, Brittany Vallejos^, Claire Wells, Carlos Cordon-cardo, Amanda Krausert, Stephanie McQuillan, Rachel Kenney, Olalekan Bello, Yu Chen, Michelle Lamendola-Essel, Hannah Mandel, Jennifer Truong, Kristen Hansen, Justin Reese, Karthik Natarajan, Jasmin Phua, Warren Kibbe, Richard Moffitt, Margaret Hall, Rishi Kamaleswaran, Jason Yoo, Anthony Solomonides, Nariman Ammar, Hannah Blau, Peter Robinson, Christopher G Chute, Ali Afshar, G C Alexander, Lisa Eskenazi, Tricia Francis, Davera Gabriel, Kirby Gong, Stephanie Hong, Harold Lehmann, Hemal Mehta, Chirag Parikh, Ann Parker, Rayna Xiao, Tanner Zhang, Richard Zhu, Jared Zook, Hythem Sidky, Christian Reich, Kristin Kostka, Brenda McGrath, Treasure Allen, Rob Schuff, Erik Benton, David A Dorr, Justin Ramsdill, Maya Choudhury, Andrew Girvin, Hannah Davis, Gina Assaf, Lisa McCorkell, Yochai Re’em, Anisha Sekar, Hannah Wei, Daniel Brannock, Rob Chew, Emily Hadley, Alexander Preiss, Ginger Tsueng, Janos Hajagos, Rachel Wong, Adit Anand, Raj Gupta, Mengyao Hu, Prahathish Kameswaran, Saarthak Kapse, Eileen Keck, Farrukh Koraishy, Spencer Krichevsky, Saaya Patel, Joel Saltz, Mary Saltz, Hiteshwar Singh, Shreya Sinha, Sam Soff, Kimon Stathakos, Chelsea Twan, Rohith Vaddavalli, Sai R Yerram, Kenneth J Wilkins, Lora Lingrey, Matvey Palchuk, Joe Flack, Andrew Williams, Robert Miller, Konstantin Kunze, Tom Best, Melissa Haendel, Aleiya Anglo, Ian Brooks, Jonathan Dekermanjian, Peter DeWitt, Tursynay Issabekova, Michael Kahn, Bryan Laraway, Sruthi Magesh, Julie McMurry, Anh Nguyen, Jenny Nguyen, Shawn T O’Neil, Meg Rebull, Chris Roeder, Seth Russell, L’tonya Starr, Anita Walden, Dave Eichmann, Charisse Madlock, Kelechi Anuforo, Ramakanth Kavuluru, Stacy Dalton, Feifan Liu, J B Byrd, Steve Johnson, Ashley Benner, Carolyn Bramante, Scott Chapman, Duy Duong, Michael Evans, Jared Huling, Corey McGee, Zheng Wang, Talia Wiggen, Rui Zhang, Jerrod Anzalone, Emily Pfaff, Monika Baskaran, Abhishek Bhatia, Namdi Brandon, Marshall Clark, Miles Crosskey, Sofia Dard, Michele Jonsson Funk, Liz Kelly, Paul Kovach, Peter Leese, Tomas McIntee, J P Powers, Til Stürmer, Kellie Walters, Michele Morris, Elaine Hill, Jack Chang, Adam Dziorny, Daniel Guth, Tanzy Love, Klint Mane, Sharad K Singh, Richa Yadav, Heidi Spratt, Jackson Barlocker, Don Brown, Sihang Jiang, Johanna Loomba, Saurav Sengupta, Suchetha Sharma, Andrea Zhou, Rena Patel, Hongfang Liu, Brian Bush, Jason Block, Tom Carton, Anna Legrand, Elizabeth Nauman, Russell Rothman, Rainu Kaushal, Sajjad Abedian, Dominique Brown, Christopher Cameron, Thomas Campion, Andrea Cohen, Marietou Dione, Rosie Ferris, Wilson Jacobs, Michael Koropsak, Alex LaMar, Colby V Lewis, Haoyang Li, Dmitry Morozyuk, Peter Morrisey, Duncan Orlander, Jyotishman Pathak, Mahfuza Sabiha, Edward J Schenck, Stephenson Strobel, Zoe Verzani, Fei Wang, Mark Weiner, Zhenxing Xu, Chengxi Zang, Yongkang Zhang, Ravi Jhaveri, L C Bailey, Christopher B Forrest, Andrea Allen, Rodrigo Azuero-Dajud, Andrew S Boss, Morgan Botdorf, Colleen Byrne, Peter Camacho, Abigail Case, Kimberley Dickinson, Susan Hague, Jonathan Harvell, Miranda Higginbotham, Kathryn Hirabayshi, Sandra Ilunga, Rochelle Jordan, Kyle Kays, Aqsa Khan, Vitaly Lorman, Nicole Marchesani, Sahal Master, Jill McDonald, Nhat Nguyen, Hanieh Razzaghi, Qiwei Shen, Alexander Shorrock, Levon H Utidjian, Ryan Webb, Kaleigh Wieand, Nate M Pajor, Jennifer Muszynski, H T Bunnell, Christopher Pennington, Cara Reedy, Yong Chen, Suchitra Rao, Parsa Mirhaji, Ravi Jhaveri, Marc Rosenman, L C Bailey, Christopher B Forrest, Hiroki Morizono, Nathan M Pajor, Soumitra Sengupta, Karthik Natarajan, W S Jones, Curtis A Kieler, Rishi Kamaleswaran, Nita N Deshpande, David Liebovitz, Vesna Mitrovic, Carol Horowitz, Patricia Kovatch, Channing Hansen, Benjamin D Horne, Thomas W Carton, Reza Shaker, Bradley Taylor, Alex Stoddard, Leslie Lenert, Katie Kirchoff, Kelly Kelleher, H T Bunnell, Daniel Eckrich, Sandra M Gonzalez, Maurice Duque, Saul Blecker, Claudia Pulgarin, Marion Sills, Erin Hickman, Dan Fort, Cynthia Chuang, Wenke Hwang, Daksha Ranade, Dimitri Christakis, Alan Schroeder, Keith E Morse, Shannon Herring, Anuradha Paranjape, Aaron Mishkin, Soledad Fernandez, Neena Thomas, Yungui Huang, Yuriy Bisyuk, Jyotsna Fuloria, Mark Pletcher, Susan Kim, Suchitra Rao, Tell Bennett, Mei Lui, Jiang Bian, Elizabeth Chrischilles, Boyd Knosp, Nick Tsinoremas, Deepthi Puram, David Williams, David Hanauer, Abu Mosa, Xing Song, Carol Geary, Jerrod Anzalone, Jonathan Arnold, Michael Becich, Srinivasan Suresh, Nickie Cappella, Richard Morgan, Asa Oxner, Athanasios Tsalatsanis, Lindsay Cowell, Phillip Reeder, Mollie Cummins, Ram Gouripeddi, Reid Holbrook, Yacob Tedla, Wei-Qi Wei, Stephen M Downs, Brian Ostasiewski, Rainu Kaushal, Thomas Campion, Katherine Irby, Kathleen Zani, Lindsay R Arthur, Ann Marie Brown, Danielle N Evans, Trenesha Hill, Laura Hobart-Porter, Lee Howard, Kathy Hummel, Hannah Krehbiel, Phaedra Yount, Amy Elliott, Grace Adam, Jyoti Angal, Maria Barber, Katelynne Clark, Clayton Dos Reis, Mandy Freesemann, Christa Friedrich, Christine Hockett, Rachel Johannsen, Emily Johnson-Vonk, Cassidy Kaiser, Alexa Kruse, Jennifer Lang, Peter Lim, Meggie McCoy, Lorie Miller, Shelby Petereit (Cerkovnik), Jaime RiChard (Werpy), Jessica Seiler, Bret Sundleaf, Joshua Svendsen, Billy Trosper, Olivia Vermeulen, Scott Young, Paul Palumbo, Sean Dabney, Marie-Christine Fahrner, Torrey Gallagher, Karilyn Martini, Mary McNally, Sarah Vivensi Stiverson, Jessica S Kosut, Venkataraman Balaraman, JoAnn Cheung, Travis K F Hong, Shanelle Kalua, Evan Minami, Andrea Siu, Micah Tong, Ronald J Teufel, Andy Atz, Marina Dantas, Tyler Kasmarcak, Kreighton Milks, Judith Ross, Chijioke Ikomi, Marisa Meyer, Gwen Pellicciotti, Thao-Ly Phan, Karen Ravin, Victoria Reynolds, Abigail Strang, Deepika Thacker, Heather Diamond, Matthew DiGuglielmo, Meg Frizzola, Annabelle Goetter, Robert Heinle, Cheyenne Katz, Karen Kowal, Shaney Pressley, Lillian Slavin, Lily Tran, Gregory Witkin, Emily Zimmerman, Genesis Agosto Roman, Akram Alshawabkeh, Ishwara Ayala Ortiz, Virginia Casey, Jose Cordero, Jocelyn De Jesus, Chrystal M Galan Rivera, Gredia Huerta-Montanez, Nilda Otero, Mayra Rivera Robles, Priscilla Roman, Genesis Roman, Zaira Rosario-Pabon, Xiodenis Santiago, Carmen Velez-Vega, Carlos Vergara, Stewart Gordon, Baylea Albarado, Tracey Allen, Allison Attuso, Taylor Ayers, Emily Bebler, Grace Bella, Alexa Bennett, John Brown, Alison Carville, Sydnie Darby, Kara Devall, Amber Dragg, Angela Elderedge, Elisabeth Fontenot, Greta Fry, Bethany Gildersleeve, Sara Goff, Lauren Harrington, Daniel S Hsia, Lisa Jones, Victoria Kaiser, Yejee Lee, Stephen Lee, Erin LeJeune, Robert Leonhard, Jennifer Levatino, Donald Lewis, Angrielle Lloyd, Ron Monce, Susannah Munro, Meghan Phillips, Blair Pucheu, Emily Rachal, Jennifer Rood, Stacey Roussel, Renee Rumsey, Connor Sanford, Monica Santos, Aryelle Stafford, Amy Thomassie, Celeste Waguespack, Katherine Walgamotte, Meredith Welch, Aubrey Windham, Michelle Stevenson, Tiffany Bell, Jackie Boyd, Soham Dasgupta, Sarah Deans, Katie Harris, Molly Hemmerle, Sarah King, Cameo McGuire, Brie Merten, Sarah Morris, Madison Ray, Brooklyn Reinhardt, Shellese Shemwell, Theresa Simeon, Katherine Walker, Sara Watson, Kathryn Weakley, Russell McCulloh, Johnathon Figliomeni, Laura Fischer, Denise Hoover, Megan Morse, Aleisha Nabower, Evan Roberts, Alice Sato, Joann Von Bon, Hengameh Raissy, David Archuleta, Rebecca Brito, Richard Campbell, Kate Cavanagh, Jude Chavez, Walter Dehority, Noella Garcia-Soberanez, Eve Gronert, Matthew Kadish, Jerry Larrabee, Debbie Lovato, Karen Luo, Noah Martinez, Analyse Merlino, Emily Reese, Sarah Ward, Kevin Wilson, Amanda Bogie, Ryan Brown, Ryan Butchee, Gina Fergeson, Ryan McKee, Brandon Mohler, Tiffany Moore, Valorie Owens, Sarah Stubbs, Timothy VanWagoner, Kelly Cowan, Meghan Bethel, Laurie Chassereau, Thomas Lahiri, Lesley Cottrell, Lauren Lake, Kathy Moffett, Emily Polak, Sarah Stutler, Charlotte Workman, David Warburton, Sindhu Mohandas, John C Wood, Emma Carpenter, Isabelle Dhindsa, Samantha Mejia, Nelly Moghadam, Candice Mulder, Sharon O’Neil, Alisha Osornio, Adrian Rios, Sydney Rosen, Andrea Smith, Deeba Tabibi, Sharon Tang, Ariana Teame, Joshua Milner, Melissa Stockwell, Brett Anderson, Tawanda Aquino, Elizabeth Berg, Steve Caddle, Marina Catallozzi, Wendy Chung, Tom Connors, Aliva De, Anny Diaz Perez, Michael DiLorenzo, Dani Dumitriu, Kanwal Farooqi, Michael Fremed, Sylvie Goldman, Kayla Kaplan, Usha Krishnan, Aimee Layton, Angela Lignelli-Dipple, Son McClaren, Jonathan Overdevest, Michelle Rodriguez, Erika B Rosenzweig, Jay Selman, Wendy Silver, Raul Silverio, Ana Valdez de Romero, Celibell Vargas, Alan Werzberger, Daniella Caputo, Camille Leggieri, Pamela Pretsch, Lawrence Kleinman, Sunanda Gaur, Johan Ayala, Arnav Batra, Lisa Cerracchio, Amber Folnagy, Maria Gennaro, Sherri Gzemski, Yue Hao, Simon Li, Yousef Masoudpour, Sandee Moroso, Manette Ness-Cochinwala, Akhil Patel, Keya Patel, Angeles Pinsa, Benjamin Richlin, Harsh Sharma, Damaris Soto, Christian Suarez, Bibiana Vargas Forero, Zain Zaidi, Lynn Olson, Kristin Davis, Alexander Fiks, Miranda Griffith, Donna Harris, Everly Macario, Jennifer Steffes, Alessandra Torres, Rangaraj Selvarangan, Dithi Banerjee, Chris Day, Kelsye Howell, Megan Mains, Juan C Salazar, William T Zempsky, Emily Bean, Carlie DeFelice, Hassan El Chebib, Katherine W Herbst, Stephanie Lesmes, Ian C Michelow, Melissa Santos, Noah Schulman, Wilson D Pace, Alicia Brooks-Greien, Karinne Colin, Christina M Hester, Ariadna Juarez-Colunga, Brian Manning, Joel Shields, Jack Westfall, Daphne York, Judy Aschner, Katharine Clouser, Justine Griswold, Donna Lee, Amanda Nowakowski, Maryellen Riordan, Hulya Bukulmez, David Kaelber, Rozina Aamir, Mohammed Abuzahrieh, Nandini Bangalore, Alexis Brown, Wendy Dalton, Suzanne Fortuna, Judi Minium, Bonnie Rosolowski, Sheila M Nolan, Amal Ahmed, Suzanne Braniecki, Montserrat Contreras, Allen Dozor, Supriya Jain, Suzanne Kaseta, Sankaran Krishnan, Zachary Messer, Armando Ramirez, Aalok Singh, Randy WIlliams, Kyung Rhee, Kelan Tantisira, Almary Akerlundh, Natacha Akshoomoff, Maria Arroyo, Wendy Barrientos, Rakesh Bhattacharjee, Bryant Y Chao, Maricela Diaz, Sergio Garcia, Sonia Garcia, Guadalupe Gomez, Trinidad Herrera, Margarita Holguin, Manaswitha Khare, Elizabeth Kiernan, Jeremy Landeo Gutierrez, Ileana Matta, Sofia Reyes, Julie Ryu, Cinthia Sanchez, Andrea Schreck, Megan R Warner, Uzma Hasan, Elizabeth Ricciardi, Vanessa Trespalacios, Vince Faustino, Andrea DeClement, Carlos R Oliveira, Kyung E Rhee, Kelan G Tantisira, Diana Acosta Valle, Almary Akerlundh, Natacha Akshoomoff, Wendy Barrientos, Rakesh Bhattacharjee, Bryant Chao, Ashvin Choudhary, Maricela Diaz, Sonia Garcia, Sergio Garcia, Maria Glenn-Arroyo, Guadalupe Gomez, Trinidad Herrera, Margarita Holguin, Manaswitha Khare, Elizabeth A Kiernan, Jeremy Landeo-Gutierrez, Anika Madan, Katelyn McConnaughey, Lisa Ramos Vallejo, Sofia Reyes, Julie Ryu, Cinthia E Sanchez, Andrea Schreck, Maira Suarez, Megan R Warner, Patricia Kinser, Amy Salisbury, Jocelyn Espinoza, Sara Moyer, Amy Rider, Sally Russell, Michael Schecter, Lindsey Stevenson, Cheryl R Stein, Stephanie V Caldas, Thomas Dylan Castro Ovalle, Anthony Chung, Orrin Devinsky, Jonathan S Farkas, Maria Isidoro-Chino, Deniz Kesebir, Eugenia Kim, Ashley Quarless, Alan Schlechter, Ranjini Srinivasan, Reina Tan, Viren D’Sa, Fatoumata Barry, Phoebe Burton, Rosa Cano Lorente, Caroline Cummins, Stephanie Wehbe, Sandra Brown, Anders Dale, Terry Jernigan, Kyung E Rhee, Susan Tapert, Jose Aguilar, David Benjamin, Natalie C Buchbinder, Norma Castro, Brandy Emerson, Hugh Garavan, Jennifer Graves, Amanda Guerrero, Janosch Linkersdoerfer, Robert Schooley, Wes Thompson, Thanh Trinh, Ron Yang, Megan Herting, Elizabeth Sowell, Cynthia Cisneros, Lauren Goedde, Cedric Manlhiot, Raul Gonzalez, Angela Laird, Jorge Limia, Robin Aupperle, Martin Paulus, Nour S El-Sabbagh, Kevin Gray, Lindsay Squeglia, Samuel Agbeh, Cori Herring, Brittany Mckenzie, Bonnie Nagel, Abby Espinoza, Anthony Hill, Angie Morales, Fiona Baker, Eva Muller-Oehring, Ingrid Durley, Mirella Dapretto, Lucina Uddin, Susan Bookheimer, Christina Caldera, Jennifer Dzul, Cinthia Zarate, Marie Banich, Paola Badilla, Jennifer Keith, G Kumar, Katie Prazak, Sara Jo Nixon, Melanie Cardoso, Meagan Sullivan, Stephen Villard, Linda Chang, Thomas Ernst, Christine Cloak, Huajun Liang, Meghann C Ryan, Mary Heitzerg, Jennifer Conley, Monica Luciana, Aaron Schroeder, Duncan Clark, Doyeon (Dan) Kim, Megan Retucci, Edward Freedman, John Foxe, Sile Ni Mhurchu, Samantha Spallina, Erin McGlade, Deborah Yurgelun-Todd, Liz Bell, Kirsten Cline, Lauren Heinrich, Perry Renshaw, Hugh Garavan, Alexandra Potter, Cass Barrett, Sofia Lozon, Samantha Spear, Christine Larson, Krista Lisdahl, Caitlin Nelson, Zach Paltzer, Bridgette Peteet, James Bjork, Mike Neale, Olive Calonge, Noah Slattery, Lisa Straub, Deanna Barch, Andrew Heath, Pamela Madden, Taylor Powell, Denise Schmitz, Dylan Gee, Zhouran (Rick) Xiang, Julie Miller, Jane Newburger, Felicia Trachtenberg, Ayesha Amarnath, James Ambrosoli, Denise Artis, Sachin Bandari, Emily Birmingham, Lozan Eyob, Kerri Hayes, Chenwei Hu, Melissa Joyce, Valentina Kazlova, Iris Liu, Amanda Marshall, Devine Mbizdenyuy, P J Mu, Robin Rowe, Brooke Sayles, Mo Zhang, Pei-Ni Jone, Michael Carr, Lauren Goodell, Wantanabe Kar, Kathleen Van’t Hof, Kristin Sexson, Elias Moussi, David Olukayode, Sandra Pena, Ricardo Pignatelli, Faridis Serrano, Sara Sexson Tejtel, Lara Shekerdemian, Audrey Dionne, Jane Newburger, Annette Baker, Sarah DeFerrantini, Thomas Giorgio, Numaira Khan, Simran Mahanta, MaryBeth Son, Matt Oster, Melissa Burnett, Kolby Sanders-Lewis, Jackie Szmuszkovicz, Fariborz Behzadian, Carla Canas, Andrew Cheng, Mike Gawad, Paige Johnson, Alicia Kazarians, Sindhu Mohandas, Anastasia Sarkissian, Brandi Scott, Consuelo Secules, Jennifer Su, Crystal Vargas, Jodie Votava-Smith, Sharon Wagner-Lees, Shuo Wang, Pierre Wong, Suchitra Rao, Sonia Chavez, Georgia Reis, Yamuna Sanil, Sanjeev Aggarwal, Aiman Almasnaah, Nirupama Kannikeswaran, Kathleen Meert, Gautam Singh, Priya Spencer, Nancy Sullivan, Sureja Sundaralingam, Vishnu Undyala, Emily Ward, Amanda Weber, Charmaine Williams Farr, Tamara Bradford, Marla Johnston, Matthew Elias, Alex Fiks, Chris Forrest, Dana Albizem, Susan Coffin, Therese Giglia, Katherine Lupton, Grace Marks, Tonia Morrison, Shawn O’Connor, Daniel Forsha, Rachel Sachdeva, Dara Watkins, Ashraf S Harahsheh, Jordyn Britton, Alix Fetch, Anita Krishnan, Onais Tariq, Sean Lang, Marisa Almaguer, Jim Cnota, Lauryn Dugan, Elise Pickering, Kathleen Rathge, Elizabeth Mitchell, Christine Capone, Nilanjana Misra, Olga Shamailova, Mark Russell, Tammy Doman, Lori Harris, Karen Hasbani, Sagar Jani, Brian McCrindle, Jessica Bainton, Maryanne Chrisant, Doris Alaby, Norma Barton, Danielle Katz, Paulette Smith, Stephanie Handler, Joe Block, Regina Cole, Jennifer Maldonado, Kim McHugh, Andrew Atz, Megan Bickford, Jason Buckley, John Costello, Delany Dennis, Elizabeth Emrath-Zwick, Mary Freeman, Lanier Jackson, Madison Johnson, Tyler Kasmarcak, Elizabeth Mack, Scott Pletzer, Natasha Ruth, Carolyn Taylor, Sinai Zyblewski, Kanwal Farooqi, Katrina Golub, Chanel Rojas, Korsin Rosalind, Chantal Sanchez, Shubhika Srivastava, Carol Prospero, Deepika Thacker, Ed Williams, Varsha Zadokar, Arash Sabati, Kylie Domian, Ashley Herzberg, Sanjana Khanna, Todd Nowlen, Susan Park, Jade Porche, Samantha Stack, Dongngan Truong, Andrea Dunn, Lilly Fagatele, Emma Joyce, Linda Lambert, Kirsten Dummer, Sherrie Bandy, Jane Burns, Katheryn Crane, Sanjeet Hedge, Joan Pancheri, Adriana Tremoulet, Ronald Mark Payne, Mary Stumpf, Michael Portman, Hidemi Kajimoto, Camden Hebson, Krissie Hock, Onyekachukwu Osakwe, Jemylle Morato, Matt Kadish, Hengameh Raissy, Jerry Larrabee, Kavita Sharma, William Anguiano, Catherine Ikemba, Alejandra Lozano, Maria Martinez, Wendy Rojas, Lerraughn Morgan, Isaura Macias, Carl Owada, Mayra Rangel, Michelle Sykes, Valerie J Flaherman, Vanessa L Jacoby, Nyat Araya, Cinthya Arellano-Mechor, Yangzom Basi, Arunee A Chang, Lauren Christopher, Isabel De La Torre, Soujanya Gade, Estefania Guerreros, Victoria Laleau, Susanne P Martin Herz, Vanessa Monzon, Yulissa Oceguera Barragan, Megumi Okumura, Michelle Rait, Marie Salem, Maria Tolentino, Torri D Metz, Jeanette P Brown, Denise Lamb, Emily Mcfarland, Amanda L Nelsen, Kim P Phillips, Shannon M Schlater, Jessica Sharma, Amber Sowles, McKaylee Zaldivar, Dwight J Rouse, Donna M Allard, Lisa M Beati, Angelica M DeMartino, Haley A Lefebvre, Jane A Milano, Emily S Miller, Matthew K Hoffman, Carrie A Kitto, Ashley Q Vanneman, Uma M Reddy, Sabine Z Bousleiman, Sara L Eccheveri, Yessenia Gutierrez, Megan M Loffredo, Andrea Perez, Rupa Ravi, Noelia M Zork, Brenna L Hughes, Jennifer W Ferrara, Monica Longo, Megan Mitchell, M S Esplin, Anna Palatnik, Mariana Karasti, Zaira Peterson, Eleanor Saffian, Samantha L Wiegand, Kathleen A Fennig, Esther K Snow, Daniel W Skupski, Rachel Pao, Ruti Patel, Honey Zaw, Beth A Plunkett, Katharine Anglemire, Constandina Kapogiannis, Katherine Kearns, Sunitha Suresh, Lynn M Yee, Mercedes Brinson, Dequana D Jones^, Michelle A Kominiarek, Gail L Mallett, Trista Reynolds, Emily Y Williams, Kristy T S Palomares, Imene Beche, Danielle Graziano-Carrete, Clara Perez, Rebecca G Clifton, Katia J Barrett, Celia M Mullowney, Grecio J Sandoval, Steven J Weiner, Kelly S Gibson, Wendy Dalton, Brittany Desantis, Parmjit Gill-Jones, Abigail Pierse, LuAnn Polito, Bonnie Rosolowski, Eugenia S Sweet, Maged M Costantine, Anna B C Bartholomew, Barbara Cackovic, Cynthia Dembroski, William A Grobman, Baylee Klopfenstein, Devra Mast, Kayla McDaniel, Melanie Paglione, Caitlin Rigsby, David N Hackney, Alan T N Tita, Donna J Armstrong, Nicole P Burrell, Brian M Casey, Donna C Dunn, Madison N Mann, M C Hoffman, Olivia Docter, Lauren M Fischer, Jocelyn Phipers, John M Thorp, Kelly W Clark, Molly A Leatherland, Sally A Timlin, Samuel Parry, Anna Filipczak, Emily Long, Meaghan G McCabe, Christina Pizzi, Hyagriv N Simhan, Evan Bauer, Jeanette E Boyce, Francesca L Facco, Sarah C Hankle, Rachel L Hines, Maura K Hohn, Ashok Muthukrishnan, Frank C Sciurba, John A Vargo, Luis D Pacheco, George R Saade, Jennifer A Cornwell, Jennifer D DeVolder, Amelia A Nounes, Ashley E Salazar, Lisa B Thibodeaux, Hector Mendez-Figueroa, Suneet P Chauhan, Felecia Ortiz, Juanita Rugerio, Jenifer Treadway, Carmen J Beamon, Inez M Dufresne, Chelsea A Grinnan, Haliey M Phillips, Christian M Pettker, Lauren Perley, Linda Rink, Teresa Akintonwa, Karima Anderson, Leyna Aragon, Mirlesna Azor-Sterlin, Bryan Bander, Leila Basu, Karyn Bishof, Frank Blancero, John Bolecek, Heather-Elizabeth Brown, Crystal Burke, Etienne Carignan, Megan Carmilani, Leah Castro Baucom, Marta Cerda, Hillary Chen, Victor Clash, Alison Cohen, Sandra Cole, Krista Coombs, Victoria Copeland, Laura Covington, Wendy Cox, Shaniese K Crawford, Hannah Davis, Felicia Davis Blakley, Alexia Debrosse, Robert DeRosa, Marissa Diggs, James Doster, Jon Douglas, Matthew Dunn, Belinda Edwards, Lia Evans, Liza Fisher, Krista Fisher, Megan Fitzgerald, Dennis Fleming, Margot Gage Witliet, Cynthia Garbutt, Tara Ghormley, Ty Godwin, Alex Gressley, Tyler Gustafson, Yvonka Hall, William (Billy) Hanlon, Teia Hassey, Verna Holmes, Mady Hornig, Maxwell Hornig, Anna Huff-Davis, Jessica Jackson Love, Nita Jain, Jennifer Jones, Geraldine Kaleponi, Jeannie Karwowsky, Christina Kim, Elijah Kindred, Jamie La Londe-Pinkston, Julie Lam, Lydia Lerma, Rebecca Letts, Juan Lewis, Joseph Lima, Doug Lindsay, Jacqui Lindsay, Heather Marti, Aaron Martinez, Christine Maughan, Lisa McCorkell, Netia McCray, Rebecca McGrath, Molly McNulty, Tony Minor, Kian Nguyen, Lauren Nichols, Elizabeth Noriega, Perla Nunes, Lisa O’Brien, Gema Ortiz, Slate Owen, Joyce Page, Aimee Peddie, Alice Perlowski, Elizabeth Phillips-Lorenzo, Lisa Prentiss, Nadia Raytselis, Lidia Regino, Megan Rockwell, Jacqueline Rutter, Robyn Saldino, Leto Sapunar, Sandria Savage, Elle Seibert, Anisha Sekar, Anita L Souder, Ezra Spier, Mallory Stanislawczyk, Doreen Stein-Seroussi, Camrynne Sullivan, Brittany Taylor, Emily Taylor, Kim Taylor, Diana Terry, Hyatt Vincent, Ann Wallace, Jess Warner, Ben White, Rochelle Wilensky, Melissa Williams, Neely Williams, Kay Williams-Dawson, Andrew Wylam, Mike Zissis

**Affiliations:** Mass General Brigham, Research Information Science and Computing, Somerville, MA, 02145, United States; Department of Public Health and Health Sciences, Bouvé College of Health Sciences, Northeastern University, Boston, MA, 02115, United States; Mass General Brigham, Research Information Science and Computing, Somerville, MA, 02145, United States; Mass General Brigham, Research Information Science and Computing, Somerville, MA, 02145, United States; Mass General Brigham, Research Information Science and Computing, Somerville, MA, 02145, United States; Mass General Brigham, Research Information Science and Computing, Somerville, MA, 02145, United States; Massachusetts General Hospital, Boston, MA, 02114, United States; Department of Population Health, NYU Langone Health, New York, NY, 10016, United States; Department of Population Health, NYU Langone Health, New York, NY, 10016, United States; Department of Population Health, NYU Langone Health, New York, NY, 10016, United States; Department of Population Health, NYU Langone Health, New York, NY, 10016, United States; Department of Population Health, NYU Langone Health, New York, NY, 10016, United States; The Feinstein Institutes for Medical Research, Northwell Health, Manhasset, NY, 11030, United States; RECOVER Patient, Caregiver, or Community Advocate Representative, New York, NY, 10016, United States; RECOVER Patient, Caregiver, or Community Advocate Representative, New York, NY, 10016, United States; RECOVER Patient, Caregiver, or Community Advocate Representative, New York, NY, 10016, United States; RECOVER Patient, Caregiver, or Community Advocate Representative, New York, NY, 10016, United States; Emory University, Atlanta, GA, 30322, United States; UNC Chapel Hill, Chapel Hill, NC, 27514, United States; Weill Cornell Medicine, Department of Population Health Sciences, New York, NY, 10065, United States; Weill Cornell Medicine, Department of Population Health Sciences, New York, NY, 10065, United States; Weill Cornell Medicine, Department of Population Health Sciences, New York, NY, 10065, United States; University of Arizona, Tucson, AZ, 85721, United States; Children’s Hospital of Philadelphia, Philadelphia, PA, 19104, United States; Department of Public Health and Health Sciences, Bouvé College of Health Sciences, Northeastern University, Boston, MA, 02115, United States; Harvard Medical School, Boston, MA, 02115, United States; Department of Medicine, Brigham and Women’s Hospital, Boston, MA, 02115, United States; Mass General Brigham, Research Information Science and Computing, Somerville, MA, 02145, United States; Massachusetts General Hospital, Boston, MA, 02114, United States; Harvard Medical School, Boston, MA, 02115, United States

**Keywords:** long COVID, PASC, EHR, patient-reported symptoms, computable phenotypes, machine learning, digital health

## Abstract

**Objective:**

Long COVID (LC) remains poorly understood, and there is a critical need for advanced computational tools to better identify and characterize patients. In this study, we use summarized symptom reports by RECOVER-Adult cohort participants linked to EHR data to characterize patients and train a computable phenotype algorithm of LC.

**Materials and Methods:**

The study included adult participants with linked Fast Health Interoperability Resource-sourced EHR data. We characterized EHR diagnoses, procedures, medications, lab tests, and vital sign features associated with LC. A computable phenotyping algorithm was trained and validated against patient-reported symptoms.

**Main Outcome and Measures:**

We assessed model discrimination and calibration in a held-out test set. We describe important model features and evaluate model discrimination and calibration.

**Results:**

The study included 1501 RECOVER-Adult cohort participants with linked EHR data. 376 (25%) met criteria for highly symptomatic LC based on the RECOVER Long COVID Research Index (LCRI). EHR features associated with LC included clinician diagnosis of shortness of breath, malaise and fatigue, and cardiac dysrhythmias; documented treatment with albuterol, gabapentin, or duloxetine; or elevated heart rate. The algorithm identifying patients with highly symptomatic LC had an area under the receiver operating characteristic curve of 0.80 (95% CI 0.74-0.85), and area under the precision-recall curve of 0.58 (95% CI, 0.47-0.69).

**Conclusion and Relevance:**

These findings demonstrate that, using EHR data, a machine-learning model can accurately select patients with sets of self-reported LC symptoms. The model could help identify patients within a health system with the highest probability of the condition and facilitate screening, recruitment for clinical trials, and etiologic studies.

## Introduction

The prolonged sequelae of COVID-19, commonly referred to as “Long COVID” (LC) or “Post Acute Sequelae of SARS-CoV-2 infection” (PASC), have emerged as a major public health challenge, affecting millions worldwide.^1^ Long COVID encompasses heterogeneous symptoms, including fatigue, brain fog, dyspnea, and neurocognitive and autonomic dysfunction.^2^ These symptoms can persist for months or years, significantly impairing health-related quality of life and complicating clinical management. Despite its widespread prevalence, the underlying pathophysiology of LC remains poorly understood, highlighting the urgent need for advanced computational tools to improve identification, characterization, and to inform research toward treatment strategies for affected individuals.

Large-scale real-world data (RWD) studies, such as those conducted by Xie et al. using Veterans Affairs (VA) EHR data, have provided valuable insights into the long-term risks associated with COVID-19.^3^ However, current EHR-based approaches often lack the granularity needed to capture the full clinical spectrum of LC. This limitation stems from inconsistent coding practices, limited use of specific diagnostic codes for LC, variability in clinician documentation, and the underrepresentation of patient-reported symptoms not routinely captured in structured fields.^4^^,^^5^

Recent efforts to operationalize LC identification at scale have made substantial progress through algorithmic phenotyping within national EHR consortia, particularly N3C and PCORnet.^6–8^ Within N3C, Pfaff et al. introduced a supervised machine-learning approach trained on patients evaluated in LC specialty clinics, later extended by Crosskey et al. to accommodate undocumented infections and reinfections through a temporally flexible modeling framework; together, these efforts demonstrated that structured EHR data can be leveraged to identify clinically coherent LC populations across diverse health systems.^7^^,^^8^ In parallel, PCORnet investigators developed transparent, rule-based phenotypes optimized for portability and interpretability, incorporating post-acute symptom and diagnosis patterns and evaluating performance against clinician chart review. Collectively, these approaches establish the feasibility, scalability, and practical utility of EHR-based LC phenotyping.^9^ At the same time, their reliance on clinician documentation and healthcare utilization as reference standards underscores an opportunity for further refinement, particularly through integration with patient-reported symptom data. The present work builds directly on this foundation by using prospectively collected symptom measures as an external reference to evaluate and inform EHR-based phenotypes, with the goal of complementing existing methods rather than replacing them.

The National Institutes of Health’s (NIH) Researching COVID to Enhance Recovery (RECOVER) Initiative represents one of the most comprehensive efforts to date to understand, diagnose, and treat LC. The RECOVER observational cohort includes more than 14 700 individuals across the United States, capturing over 50 patient-reported symptoms such as fatigue, brain fog, and shortness of breath.^10^ A key strength of the RECOVER study is its prospective collection of both patient-reported and clinical data collected through regular follow-ups, creating an invaluable dataset for the development of more precise, symptom-driven phenotyping algorithms. This integration of detailed EHR data with patient-reported outcomes provides a unique opportunity to advance LC research, enabling the creation of phenotypes that more accurately reflect the diverse and evolving nature of the condition.

In this study, we use symptom reports by RECOVER-Adult participants linked to EHR data using an innovative digital health platform to characterize patients and create and validate a computable phenotype algorithm of LC.

## Methods

### Population and data source

We used data from the RECOVER-Adult study. RECOVER-Adult is a prospective, longitudinal cohort study involving adults across the United States and Puerto Rico enrolled between October 2021 and December 2022. This study aims to understand the prevalence, symptoms, risk factors, and biological mechanisms of LC.^10^ Participants were enrolled both with and without prior SARS-CoV-2 infection and included a small subset of individuals recruited while pregnant through the RECOVER pregnancy cohort. Participants undergo regular assessments, including surveys, physical exams, and laboratory tests. Participants are recruited from academic medical centers, community outreach, public health agencies, or self-referral and are demographically representative of the overall US population.

**Figure 1.**
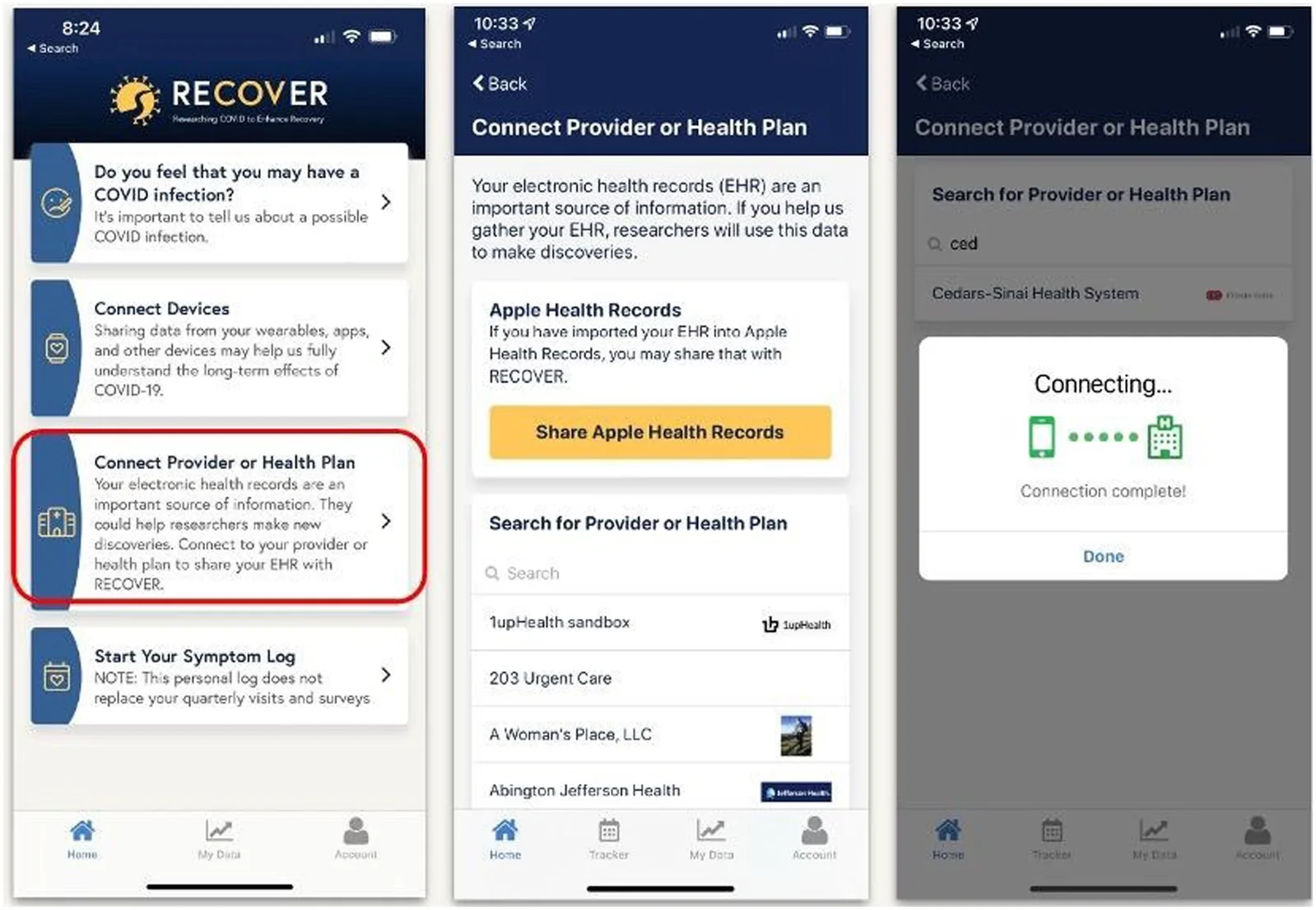
Depiction of RECOVER Digital Health Platform mobile application participant workflow to authorize electronic health record data transfer from their healthcare provider or health plan to the RECOVER Data Resource Core via the FHIR messages.

RECOVER-Adult participants are also encouraged to enroll in the RECOVER Digital Health Program to submit their EHR data by authorizing data transfer from the healthcare provider’s patient portal via Fast Health Interoperability Resource (FHIR).^11^ EHR data transferred includes retrospective data available in healthcare provider patient portals (as far back as the 1970’s in a few cases, see Figure S1 for data density by time of EHR data). Upon establishing the initial connection, EHR data continued to be transmitted through April 7, 2025 to the RECOVER Data Resource Core (DRC) as patients have subsequent visits with their healthcare providers (Figure 1). EHR data is transferred to the DRC and transformed to the Observation Medical Outcomes Partnership common data model.^12^ The data is then linked with observational cohort data using integrating informatics for the bench to bedside (i2b2) software.^13^

Sociodemographic data is self-reported by participants at enrollment and includes age, sex at birth, education level, race, and ethnicity. The index (first) SARS-CoV-2 infection is obtained by positive laboratory testing, symptom reports, and/or clinician diagnosis. The timeframe of index infection is divided into pre-Omicron (prior to December 1, 2021) and Omicron. Home residence at enrollment is used to identify participants who live in rural areas and medically underserved areas with diminished access to care.

The RECOVER-Adult study and Digital Health Platform were approved by the NYU Langone and Mass General Brigham institutional review boards. All participants provided informed consent for prospective study participation and EHR data contribution.

### Patient-reported Long COVID symptom measurements

RECOVER-Adult participants complete LC symptom surveys at 3-month intervals after enrollment. The instruments include questions about 57 LC-related symptoms and severity scales.^10^ The RECOVER Long COVID Research index (LCRI) is a measure for provisional, research-based classification of LC patients at 6 months or more after index date of infection based on participant symptom reports at the first post-acute visit of 11 machine learning-derived symptoms, including: post-exertional malaise, fatigue, brain fog, dizziness, palpitations, change in smell or taste, thirst, chronic cough, chest pain, shortness of breath, and sleep apnea.^14^^,^^15^ The participant-reported symptoms included in the LCRI were more common in participants with known SARS-CoV-2 infection compared to participants without known prior SARS-CoV-2 infection. Individual symptoms contribute variable point values based on their relative association with LC status. Participants with a score of 11+ were classified as “highly symptomatic Long COVID.” Participants with a score between 1 and 10 were classified as “Possible LCRI symptom.” Participants with 0 symptoms in the LCRI were classified as “No LCRI symptom.” We classified participants into highly symptomatic LC if the LCRI was ≥11 at any follow-up time point 3-48 months after the index date of infection. We classified patients with a summarized LCRI symptom index <11 as controls for characterization and phenotyping purposes. A secondary analysis groups participants with an LCRI > 0 as cases and LCRI = 0 as controls.

### EHR feature selection and engineering

EHR data is high-dimensional data with over 200 000 distinct concepts in the study dataset. Five domains of codified data, including diagnosis, procedures, medications, abnormal lab measurements, and vital signs were grouped into semantically related concepts as described in Chuan et al.^16^ International Classification of Disease—Clinical Modification 9 and 10 (ICD-9-CM and ICD-10-CM) and Systematized Medical Nomenclature for Medicine (SNOMED) codes were aggregated into PheCodes to represent clinical diagnosis groups using mapping version 1.2 (https://phewascatalog.org/phecodes).^17^ PheCode labels in tables and figures are used as provided in the source vocabulary. For procedure codes, including CPT-4, HCPCS, ICD-9-PCS, ICD-10-PCS, we assigned clinical classification software (CCS) categories based on the CCS mapping (https://www.hcup-us.ahrq.gov/toolssoftware/ccs_svcsproc/ccssvcproc.jsp).^18^ For medications, we aggregated medication codes to ingredient-level RxNorm codes.^19^ Lab tests and vital signs were mapped to LOINC codes.^20^^,^^21^ Because there was significant variation of lab coding, units, and result format, informaticians (A.C., V.M.C.) manually curated 32 lab and vital results and constructed features for high, low, or abnormal labs. Abnormal flags were determined using reference ranges included in the FHIR payload from source labs.

For each participant feature, we computed the count of distinct days when the feature was recorded at any time in the EHR through April 2025. We did not anchor features to SARS-CoV-2 infection timing due to incomplete and variable capture of infection dates in EHR data, particularly with increased use of at-home testing during later pandemic phases. While index infection is available in the clinical cohort data, incorporating this date in feature construction would limit generalizability of our model to other datasets, a primary aim of this work.

Feature counts were log-transformed (log1p) and features occurring in fewer than 5 people in the entire study data set were excluded. The final feature space includes participant age at enrollment, sex at birth, total number of visits, number of inpatient encounters, and a total of 941 EHR feature groups that passed frequency control.

### Model training

The dataset was divided into a model training (*N* = 1201; 75%) and testing (*N* = 376; 25%) set, with random assignment stratified by outcome status to ensure an equivalent representation of LC status in each set. We trained a regularized logistic regression model using the Least Absolute Shrinkage and Selection Operator (LASSO) in the training set. LASSO performs variable selection and regularization to enhance the prediction accuracy and interpretability of models. The primary model incorporated age, sex at birth, number of visits, and all EHR features that met the frequency cutoff, including diagnosis, medications, procedures, and normal and abnormal laboratory tests and vital signs. To improve the accuracy of the model, we used 10-fold cross-validation to identify the optimal combination of parameters that resulted in the best overall performance on the training data. Modeling and evaluation were conducted in R 4.4.2 using the glmnet and uniLasso libraries.^22^^,^^23^

### Long COVID clinician documentation reference model

ICD-10-CM code U09.9 for LC, titled “Post COVID Conditions,” was introduced in October 2021. Coding guidelines from the National Center for Health Statistics (NCHS) state that this code should be used as a secondary diagnosis to indicate LC when the patient has a history of confirmed or suspected COVID-19 and presents with persistent or new health issues attributable to the prior infection.^24^ ICD-10-CM U09.9 is commonly used for LC cohort selection in RWD studies and has been used to inform the training of EHR-only computable phenotype models.^8^^,^^9^ To evaluate performance of direct clinician documentation in our study cohort, we built a single feature model with the count of U09.9 codes and evaluated the accuracy and sensitivity of at least 1 code in the participants’ EHR data. Prior to the introduction of the U09.9 code, clinicians used the code B94.8 titled “sequelae of infectious and parasitic diseases,” to document LC symptoms. We also evaluate the performance of both the older and newer codes in our comparison to the trained computable phenotype model.

### Model evaluation and statistical analysis

For both the training and testing sets, model discrimination was assessed by the area under the receiver operating characteristic curve (AUROC), sensitivity, and positive and negative predictive values (PPV, NPV) using a screen-positive threshold at a specificity of 95% in the training set. Similarly, calibration was assessed by area under the precision-recall curve (AUPRC), the Brier class score and by comparing observed, expected event rates by risk decile calibration plots. The Brier score measures calibration by quantifying the average squared difference between predicted probabilities and actual outcomes, where lower values indicate better calibration.^25^ Finally, we compared the primary model’s statistical parity in the test set among the subgroups defined by participants’ sex at birth, race and ethnicity, age at study enrollment (categorized as 18-44, 45-65, and greater than 65 years), prevalent SARS-CoV-2 variant at index infection (pre-Omicron prior to December 1, 2021 or Omicron occurring on or after December 1), and participants residing in a medically underserved area to evaluate model performance in these different groups of people. 95% CI for performance metrics were computed with 1000 bootstrap samples with replacement in the test set. Model discrimination and calibration were also assessed in a secondary model with any LC symptom (RECOVER LCRI ≥1). We followed the TRIPOD reporting guideline for model development and validation.^26^

## Results

**Figure 2.**
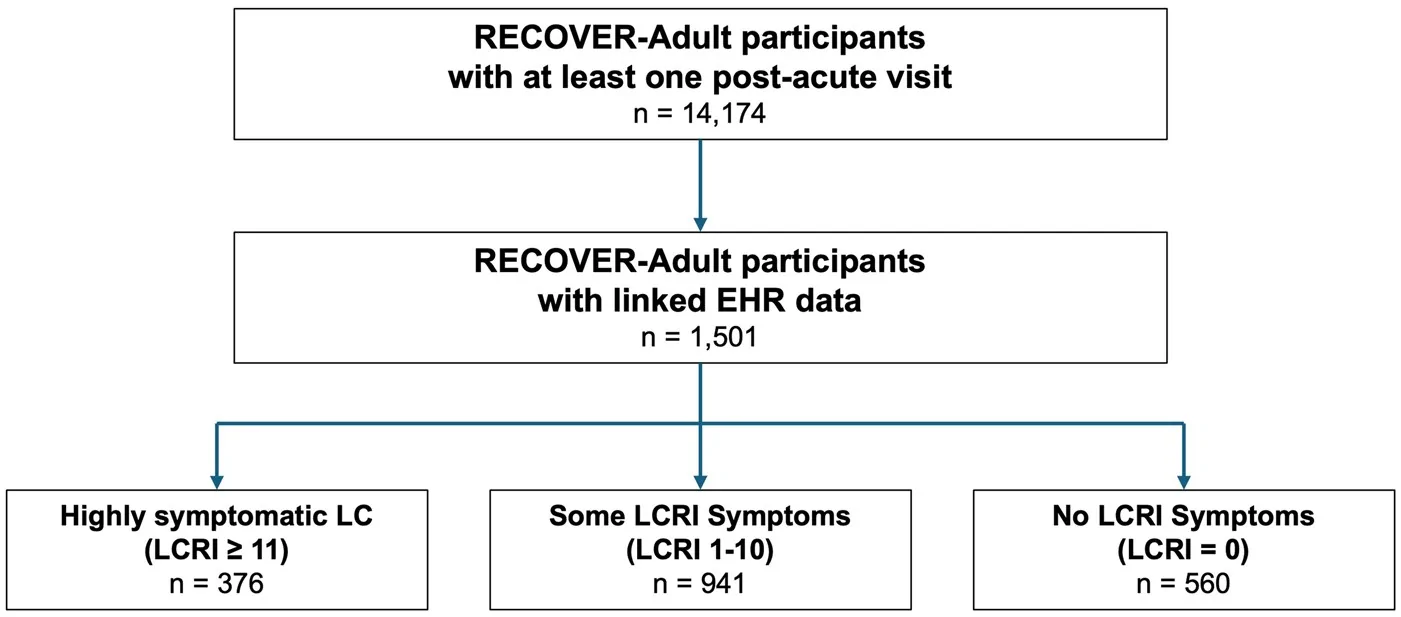
Study inclusion diagram with Long COVID status in the study cohort. LC: Long COVID.

Of 14 172 RECOVER-Adult participants with a study visit 3 months or more after the index date, 1501 contributed EHR data (11%). Table 1 summarizes the demographic and clinical characteristics of study individuals with highly symptomatic Long COVID (LCRI ≥ 11) compared to those with some or no Long COVID symptoms (LCRI < 11). Among all patients, the median age was 46 years [interquartile range (IQR) 36-59]. 1082 (72%) were female at birth and 75 (5.1%) self-identified as non-Hispanic Asian, 90 (6.0%) as non-Hispanic Black, 1099 (73%) as non-Hispanic White, 162 (11%) as Hispanic, and 74 (4.9%) as multiple races or another race not otherwise classified. At enrollment, 339 (23%) participants resided in a medically underserved area and 85 (5.7%) in a rural area. 472 (31%) individuals had an index SARS-CoV-2 infection before the Omicron variant was prevalent (before December 1, 2021) and 1029 (69%) during the Omicron-prevalent period. 376 participants (25%) met criteria for highly symptomatic Long COVID with LCRI > 11 (Figure 2). A comparison of participants contributing EHR to all other RECOVER-Adult participants is presented in Table S1. Participants contributing EHR data were more likely to be older, White, Non-Hispanic, and have a bachelor’s degree or above.

**Table 1.** Sociodemographic characteristics of RECOVER-Adult participants contributing EHR data comparing individuals with highly symptomatic long COVID (LCRI ≥ 11) to individuals with possible or no Long COVID symptoms (LCRI < 11).

Characteristic	**Overall** *N* = 1501	**Highly symptomatic LC** (LCRI ≥ 11) *N* = 376	**Possible/No LC** (LCRI < 11) *N* = 1125
Female sex at birth, *n* (%)	1082 (72)	300 (80)	782 (70)
Age at enrollment, y, Median (IQR)	46 (36-59)	46 (38-57)	46 (35-61)
Age category at enrollment, y, *n* (%)			
18-45	740 (49)	188 (50)	552 (49)
46-65	540 (36)	155 (41)	385 (34)
>65	221 (15)	33 (8.8)	188 (17)
Race/ethnicity, *n* (%)			
Hispanic	162 (11)	44 (12)	118 (10)
Mixed race/Other/Missing	74 (4.9)	23 (6.1)	51 (4.5)
Non-Hispanic Asian	76 (5.1)	13 (3.5)	63 (5.6)
Non-Hispanic Black	90 (6.0)	16 (4.3)	74 (6.6)
Non-Hispanic White	1099 (73)	280 (74)	819 (73)
Prevalent SARS-CoV-2 strain at index date, *n* (%)			
Omicron	1029 (69)	156 (41)	873 (78)
pre-Omicron	472 (31)	220 (59)	252 (22)
Education level, *n* (%)			
Did not complete high school/no diploma	5 (0.3)	2 (0.5)	3 (0.3)
High school/GED/some college/vocational/technical	374 (25)	128 (34)	246 (22)
Bachelor’s/advanced degree	1116 (75)	244 (65)	872 (78)
Rural participant, *n* (%)	85 (5.7)	35 (9.3)	50 (4.4)
Medically underserved area, *n* (%)	339 (23)	96 (26)	243 (22)

Among the 1501 study participants, 89 (5.9%) had at least one clinician-documented diagnosis of Long COVID (ICD-10-CM U09.9) recorded in their medical record, yielding a sensitivity of 0.15 and a positive predictive value (PPV) of 0.64 for identifying individuals with highly symptomatic Long COVID (LCRI ≥ 11). When the case definition was broadened to include possible pre–October 2021 diagnoses—captured by either ICD-10-CM U09.9 (LC) or B94.8 (sequelae of infectious disease)—191 participants (12.7%) met this criterion. Under this expanded definition, the sensitivity and PPV were 0.34 and 0.67, respectively, for participants with LCRI ≥ 11, and 0.19 and 0.91, respectively, for participants with LCRI ≥ 1.

The top diagnoses associated with highly symptomatic Long COVID status after adjusting for age and sex were shortness of breath, malaise and fatigue, infectious and parasitic diseases, cardiac dysrhythmias, respiratory symptoms, and myalgic encephalomyelitis/chronic fatigue syndrome (ME/CFS) (including ICD-10 G93.32). The top medications were albuterol, gabapentin, duloxetine, budesonide, fluticasone, and prednisone. Top procedures include electroencephalogram (EEG), echocardiography, and diagnostic cardiac catheterization. Top associated measurements include normal C-reactive protein level, elevated heart rate, low potassium level, and both normal ferritin level and elevated ferritin level. Table 2 includes the top features for each data domain and Figure S2 is a Manhattan plot of all EHR features.

**Table 2.** Top EHR Features associated with highly symptomatic Long COVID (LCRI ≥ 11) as compared with no/possible Long COVID (LCRI < 11).

EHR feature	OR	SE	*P*-value	**Highly Symptomatic LC** (LCRI ≥ 11) *N* = 376	**Possible/No LC** (LCRI < 11) *N* = 1125
**Diagnosis**					
PheCode 512.7—Shortness of breath	4.73	0.13	2.72 × 10^−33^	198 (53)	217 (19)
PheCode 798—Malaise and fatigue	4.17	0.13	9.56 × 10^−30^	234 (62)	318 (28)
PheCode 136—Other infectious and parasitic diseases	6.01	0.17	8.09 × 10^−27^	110 (29)	73 (6.5)
PheCode 427—Cardiac dysrhythmias	3.34	0.13	1.61 × 10^−21^	187 (50)	260 (23)
PheCode 512—Other symptoms of respiratory system	2.94	0.13	1.21 × 10^−17^	250 (66)	458 (41)
PheCode 798.1—Chronic fatigue syndrome (ME/CFS)	7.46	0.24	1.11 × 10^−16^	59 (16)	27 (2.4)
PheCode 770—Myalgia and myositis unspecified	5.24	0.20	4.14 × 10^−16^	71 (19)	45 (4.0)
PheCode 292—Neurological disorders	3.31	0.15	9.74 × 10^−16^	110 (29)	123 (11)
PheCode 350—Abnormal movement	4.30	0.19	3.33 × 10^−15^	75 (20)	64 (5.7)
ICD-10-CM U09.9—Post COVID19 Condition	6.12	0.23	5.26 × 10^−15^	57 (15)	32 (2.8)
PheCode 427.7—Tachycardia NOS	3.74	0.17	1.11 × 10^−14^	85 (23)	81 (7.2)
PheCode 339—Other headache syndromes	2.67	0.13	1.26 × 10^−14^	164 (44)	246 (22)
PheCode 327.3—Sleep apnea	2.78	0.13	3.04 × 10^−14^	148 (39)	238 (21)
PheCode 427.9—Palpitations	2.80	0.14	2.38 × 10^−13^	120 (32)	160 (14)
PheCode 327.32—Obstructive sleep apnea	2.67	0.14	2.03 × 10^−12^	129 (34)	206 (18)
PheCode 338—Pain	2.58	0.13	2.36 × 10^−12^	135 (36)	203 (18)
PheCode 386.9—Dizziness and giddiness	4.63	0.22	5.42 × 10^−12^	53 (14)	39 (3.5)
PheCode 327—Sleep disorders	2.34	0.13	1.59 × 10^−11^	174 (46)	321 (29)
PheCode 799—Debility unspecified	3.65	0.20	3.51 × 10^−11^	62 (16)	58 (5.2)
PheCode 296—Mood disorders	2.22	0.13	2.49 × 10^−10^	161 (43)	280 (25)
**Medications**					
RXNORM 435—albuterol	3.17	0.13	3.35 × 10^−20^	244 (65)	412 (37)
RXNORM 25480—gabapentin	2.98	0.13	8.45 × 10^−17^	159 (42)	228 (20)
RXNORM 72625—duloxetine	3.57	0.16	4.26 × 10^−15^	93 (25)	93 (8.3)
RXNORM 19831—budesonide	3.36	0.16	1.66 × 10^−13^	87 (23)	94 (8.4)
RXNORM 41126—fluticasone	2.40	0.12	7.20 × 10^−13^	208 (55)	380 (34)
RXNORM 8640—prednisone	2.29	0.12	2.50 × 10^−11^	178 (47)	316 (28)
RXNORM 30125—modafinil	5.70	0.27	6.88 × 10^−11^	41 (11)	24 (2.1)
RXNORM 6918—metoprolol	2.68	0.15	1.80 × 10^−10^	95 (25)	133 (12)
RXNORM 7646—omeprazole	2.31	0.13	2.26 × 10^−10^	137 (36)	222 (20)
RXNORM 187832—pregabalin	3.78	0.21	4.12 × 10^−10^	52 (14)	48 (4.3)
RXNORM 4278—famotidine	2.22	0.13	5.16 × 10^−10^	150 (40)	251 (22)
RXNORM 25255—formoterol	3.11	0.19	2.12 × 10^−9^	62 (16)	66 (5.9)
RXNORM 40114—guanfacine	5.42	0.29	4.21 × 10^−9^	34 (9.0)	21 (1.9)
RXNORM 11248—vitamin B12	2.65	0.17	8.33 × 10^−9^	75 (20)	97 (8.6)
RXNORM 6809—metformin	2.52	0.16	1.02 × 10^−8^	82 (22)	114 (10)
RXNORM 7243—naltrexone	3.83	0.24	1.33 × 10^−8^	42 (11)	37 (3.3)
RXNORM 7213—ipratropium	2.49	0.16	1.35 × 10^−8^	82 (22)	114 (10)
RXNORM 40790—pantoprazole	2.24	0.14	1.38 × 10^−8^	109 (29)	174 (15)
RXNORM 6963—midodrine	6.97	0.35	3.09 × 10^−8^	29 (7.7)	12 (1.1)
RXNORM 1191—aspirin	2.12	0.14	5.01 × 10^−8^	123 (33)	225 (20)
**Procedures**					
CCS 38 Other diagnostic procedures	2.58	0.16	5.76 × 10^−9^	80 (21)	109 (9.7)
CCS 199 EEG	5.77	0.35	3.98 × 10^−7^	24 (6.4)	14 (1.2)
CCS 193 Echocardiography	1.97	0.15	5.83 × 10^−6^	91 (24)	161 (14)
CCS 47 Diagnostic cardiac catheterization	4.77	0.41	1.34 × 10^−4^	15 (4.0)	11 (1.0)
CCS 7 Other diagnostic nervous	1.93	0.18	3.46 × 10^−4^	55 (15)	93 (8.3)
CCS 203 Electrographic cardiac monitoring	2.26	0.23	3.73 × 10^−4^	36 (9.6)	50 (4.4)
CCS 220 Ophthalmologic and otology	0.59	0.18	3.30 × 10^−3^	43 (11)	205 (18)
CCS 208 Radioisotope pulmonary scan	2.79	0.35	3.76 × 10^−3^	16 (4.3)	18 (1.6)
CCS 30 Tonsillectomy and or adenoidectomy	3.89	0.47	3.86 × 10^−3^	11 (2.9)	8 (0.7)
CCS 201 Stress tests	2.31	0.29	4.38 × 10^−3^	21 (5.6)	31 (2.8)
CCS 182 Mammography	0.47	0.31	1.55 × 10^−2^	13 (3.5)	68 (6.0)
CCS 179 CT scan, abdomen	1.73	0.25	2.86 × 10^−2^	27 (7.2)	50 (4.4)
CCS 218 Psychological and psychiatric procedure	1.82	0.30	4.29 × 10^−2^	19 (5.1)	34 (3.0)
CCS 84 Cholecystectomy & duct exploration	2.05	0.38	6.16 × 10^−2^	12 (3.2)	17 (1.5)
**Labs and vitals**					
CRP—Normal	2.21	0.12	2.01 × 10^−10^	244 (65)	513 (46)
Heart rate—High	2.04	0.13	1.42 × 10^−8^	181 (48)	348 (31)
Ferritin—Normal	1.81	0.12	1.22 × 10^−6^	177 (47)	364 (32)
Potassium—Low	2.03	0.15	1.25 × 10^−6^	99 (26)	163 (14)
Ferritin- High	2.93	0.23	3.60 × 10^−6^	39 (10)	43 (3.8)
Troponin I—Normal	1.74	0.12	4.48 × 10^−6^	207 (55)	470 (42)
Creatinine—High	2.02	0.16	9.45 × 10^−6^	81 (22)	148 (13)
CRP—High	1.71	0.12	1.44 × 10^−5^	162 (43)	341 (30)
Hemoglobin—High	2.13	0.18	3.59 × 10^−5^	57 (15)	89 (7.9)
BMI—High	1.59	0.12	1.12 × 10^−4^	202 (54)	464 (41)
Calcium—Low	1.71	0.14	1.17 × 10^−4^	107 (28)	211 (19)
Bilirubin—Normal	2.13	0.20	1.22 × 10^−4^	342 (91)	929 (83)
D dimer—Normal	1.58	0.12	1.58 × 10^−4^	203 (54)	476 (42)
SBP—Normal	2.28	0.22	2.42 × 10^−4^	351 (93)	959 (85)
HR—Normal	1.96	0.19	3.16 × 10^−4^	337 (90)	909 (81)
Urea nitrogen—Normal	1.97	0.19	4.40 × 10^−4^	340 (90)	931 (83)
DBP—Normal	2.39	0.25	4.53 × 10^−4^	356 (95)	985 (88)
Chloride—Normal	1.96	0.19	5.00 × 10^−4^	340 (90)	933 (83)
Alkaline phosphatase—Normal	1.89	0.18	5.01 × 10^−4^	335 (89)	915 (81)
Sodium—Low	1.61	0.14	7.06 × 10^−4^	102 (27)	213 (19)

The top features for each EHR data domain (diagnosis, procedures, medications, lab tests and vital signs) are shown. Features are ranked by lowest *P*-value for models adjusted for age and sex at birth.

**Figure 3.**
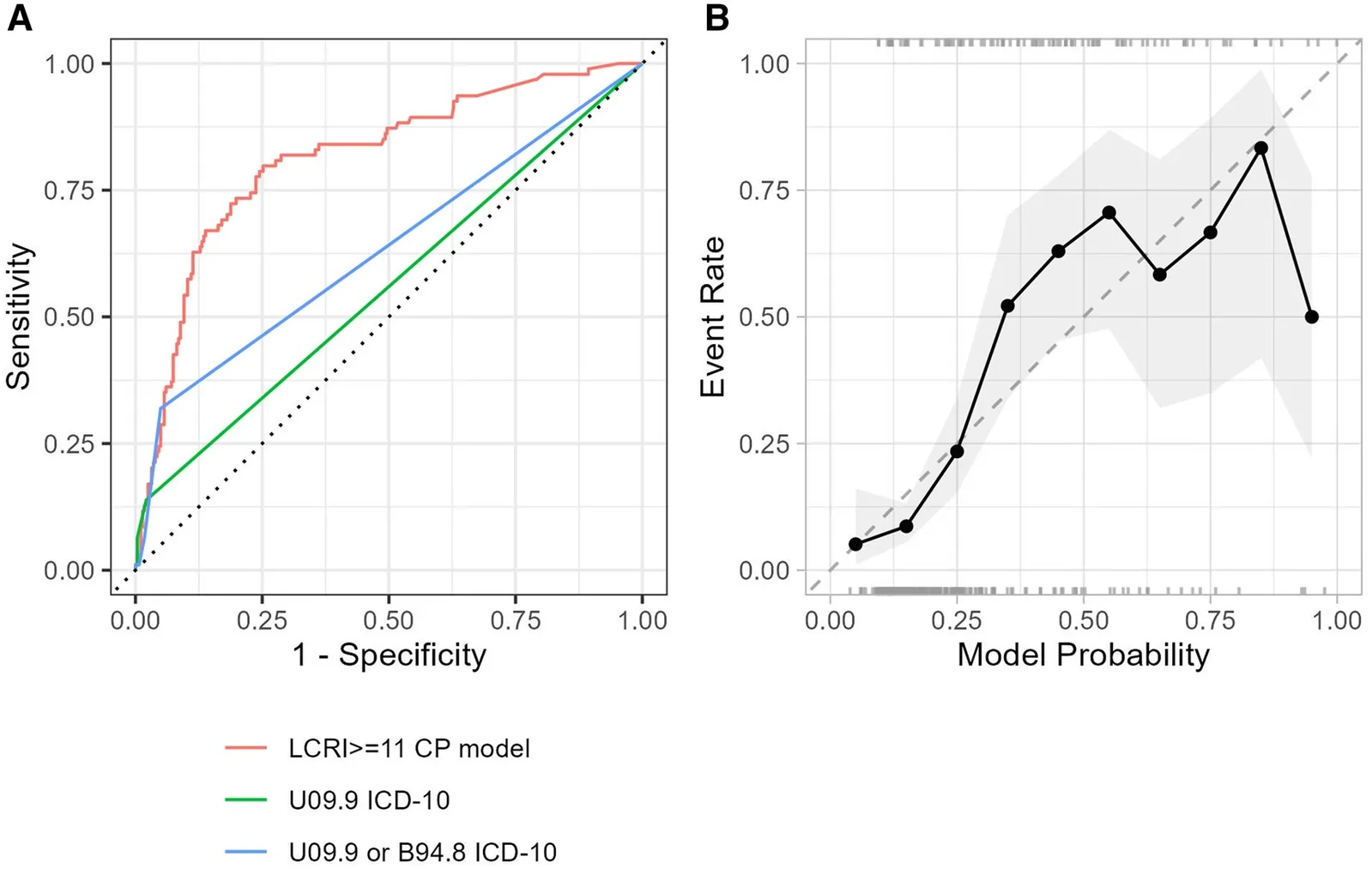
(A) Receiver operating characteristic plot of the machine learning computable phenotyping model, ICD-10-CM U09.9 Post COVID-19 conditions clinician diagnosis code, or a combination of ICD-10-CM U09.9 and B94.8 sequelae of infectious disease; and (B) Calibration plot of the machine learning model.

In the testing dataset, the highly symptomatic Long COVID model (LCRI ≥ 11) achieved an AUROC of 0.80 (95% CI, 0.74-0.85), AUPRC of 0.58 (95% CI, 0.47-0.69), and a Brier score of 0.14 (95% CI, 0.12-0.17), indicating good discrimination and calibration (Figure 3A and B, Table 3). The top 5 weighted predictors were EEG, cefadroxil prescriptions, diagnoses of infectious and parasitic diseases, painful respiratory symptoms, and diagnoses of Post-COVID-19 conditions (U09.9) (Figure S2). At 95% specificity, PPV was 65% (95% CI, 53%-76%), NPV was 84% (95% CI, 79%-88%), and sensitivity was 47% (95% CI, 37%-58%) (Table S3). The secondary phenotype model (LCRI ≥ 1) had lower discrimination but improved calibration (AUROC 0.69, 95% CI, 0.64-0.74; AUPRC 0.82, 95% CI, 0.76-0.87; Brier score 0.21, 95% CI, 0.19-0.23). Lower discrimination of the LCRI ≥ 1 model likely reflects increased heterogeneity and reduced label specificity rather than diminished signal in EHR features.

**Table 3.** Performance metrics of machine learning model identifying patients with highly symptomatic LC (LCRI ≥ 11) based on EHR features.

Subgroup		*N*	AUROC	95% CI LL	95% CI UL	AUPRC	95% CI LL	95% CI UL
**Overall**	**All participants in test se**t	**376**	**0.80**	**0.74**	**0.85**	**0.58**	**0.47**	**0.69**
Sex at birth	Male	110	0.80	0.67	0.90	0.59	0.46	0.71
	Female	266	0.79	0.73	0.86	0.59	0.38	0.77
Age at enrollment	18-45	192	0.83	0.76	0.90	0.69	0.55	0.80
	46-65	116	0.79	0.68	0.88	0.45	0.14	0.73
	>65	68	0.67	0.48	0.83	0.56	0.40	0.74
Medically underserved	No	309	0.79	0.72	0.85	0.49	0.32	0.70
	Yes	67	0.80	0.69	0.91	0.64	0.52	0.75
Prevalent SARS-CoV-2 variant at index infection	Omicron	248	0.80	0.71	0.89	0.43	0.28	0.59
	pre-Omicron	128	0.75	0.66	0.84	0.70	0.56	0.83
Race/ethnicity	Non-Hispanic White	273	0.78	0.70	0.84	0.47	0.04	1.00
	Hispanic	48	0.88	0.76	0.97	0.64	0.52	0.75
	Non-Hispanic Black	20	0.94	0.78	1.00	0.72	0.15	1.00
	Non-Hispanic Asian	15	0.64	0.15	1.00	0.41	0.20	0.70
	Mixed race/Other/Missing	20	0.85	0.64	1.00	0.59	0.08	1.00

Metrics are displayed for the held-out testing set only and are reported for the entire set and for select subgroups to evaluate demographic parity for discrimination and calibration of the model.

Abbreviations: AUPRC, area under the precision-recall curve; AUROC, area under the receiver operating curve; CI, confidence interval, LL, lower limit, UL, upper limit.

Finally, we assessed model fairness and performance parity by examining model discrimination (AUROC) and calibration (AUPRC) by subgroups of sex, age, race and ethnicity, and participants residing in medically underserved and prevalent SARS-CoV-2 variant at index infection (pre-Omicron prior to December 1, 2021, or Omicron occurring on or after December 1). Results are included in Table 3, and threshold metrics are shown in Table S3. In general, the model performed similarly in all groups with a small degradation in performance for participants older than 65 years.

## Discussion

In this US-wide study of over 1500 RECOVER participants with linked EHR data, 40% reported at least one LC-related symptom, and 25% met the criteria for highly symptomatic Long COVID based on the RECOVER LCRI. Structured EHR diagnoses associated with highly symptomatic Long COVID included malaise and fatigue, shortness of breath, and infectious diseases. Medications associated with LC status included albuterol, gabapentin, and duloxetine.

A machine learning model trained on clinician-recorded EHR codes demonstrated strong discrimination and calibration in identifying LC cases defined by symptoms, achieving an AUROC of 0.80 and an AUPRC of 0.58 in an independent test set. This phenotyping approach is one of the few EHR models validated using prospectively collected patient-reported symptom data.

In contrast, reliance on a single structured diagnosis code for Post COVID-19 conditions demonstrated poor sensitivity. Fewer than 16% of RECOVER-adult participants with highly symptomatic LC had the clinician-documented code in their EHR. This finding aligns with prior research in large EHR-based cohorts.^27^^,^^28^ The undercoding of LC, ME/CFS, and other LC symptoms may stem from several factors, including lack of clinician awareness, clinician uncertainty, limited access to care, and billing practices that do not prioritize LC diagnoses. It may also represent overreliance on assignment of diagnoses in the neuropsychiatric domain, such as disorders of mood and sleep.^29^

Our findings support the potential of EHR data to accurately identify patients with self-reported LC symptoms. Despite the undercoding of LC diagnoses, a computable phenotype algorithm integrating demographics, symptoms, diagnoses, medications, procedures, laboratory results, and vital signs demonstrated high discrimination and calibration.

This study also highlights a novel approach to integrating EHR data, leveraging the FHIR data standard and a participant-led, app-based authorization process within the RECOVER Digital Health Platform. Unlike traditional research cohorts that rely on batch data submissions from study sites, the FHIR-based approach empowers participants to directly control and share their EHR data.^30^^,^^31^ This methodology enhances data completeness by allowing contributions from diverse healthcare and insurance providers beyond those affiliated with the study.

Several limitations should be considered. First, the study cohort may not fully represent the broader population of LC patients. Participation in the RECOVER Digital Health Program required patient portal access and authorization, which may preferentially include individuals with greater digital access, health system engagement, and trust in data sharing, introducing selection bias beyond the underlying RECOVER cohort. While RECOVER-Adult study enrollment reflects the demographic diversity of the U.S., the cohort is more educated than the average population and the subset of participants contributing EHR data exhibits lower socioeconomic diversity. Additionally, this analysis focuses exclusively on adults, limiting generalizability to pediatric populations. These factors may limit transportability of the model to underserved populations and highlight the need for external validation in more diverse real-world settings prior to deployment.

Second, the phenotyping approach does not incorporate the temporality of EHR data, which simplifies model implementation but may obscure important trends. Identifying an index date of SARS-CoV-2 infection in EHRs is particularly challenging due to changes in testing practices over time. Furthermore, clinical features associated with LC may represent either causal factors or downstream effects of the condition. As a result, features may reflect care patterns and healthcare utilization associated with LC rather than temporally proximal predictors and should be interpreted accordingly. DHP records included retrospective longitudinal EHR data that could precede RECOVER enrollment by several years, some model features may additionally reflect pre-existing comorbidities and historical healthcare utilization patterns rather than post-COVID changes alone. Future work should focus on identifying subphenotypes of LC and exploring causal inference techniques to refine feature selection and develop a LC prediction model.

Third, this study includes only internal validation, and external validation in independent healthcare system datasets is required to establish generalizability and transportability. We have developed an open-source R package, longcovidcp, which enables computation of the phenotype score in external datasets using standardized vocabularies and feature mappings.^32^ We hope this resource will facilitate independent validation, benchmarking against other LC phenotyping approaches, and evaluation across healthcare systems with differing patient populations and documentation practices.

Lastly, in this study we developed and validated a computable phenotype algorithm of LC that was referenced against patient-reported symptom data that yielded LCRI scores with a range of likelihood for LC. Considering that the LCRI score continues to undergo iterative refinements for what is an evolving research definition, the computable phenotype will need to undergo further refinements to maintain test characteristics. We, therefore, must caution the application of this research tool for routine clinical care or denial of benefits for fear of missing monosymptomatic presentations of LC. Despite this, our computable phenotype performed better than clinician-diagnosed LC (measured as ICD10 U0.9.9 code); hence, application in clinical circumstances for the identification of cases may need to be contextualized to settings with low case detection rates. Importantly, computable phenotypes should be interpreted as a probabilistic tool for population-level identification rather than a diagnostic instrument for individual patients. From a clinical perspective, highly ranked features reflect patterns of care and documentation associated with symptom burden, not necessarily causal mechanisms or disease-defining markers.

Despite these limitations, our approach provides a robust framework for cohort identification in LC research. The computable phenotype algorithm can facilitate clinical trial recruitment, epidemiological analyses, and assessments of healthcare utilization patterns among patients with LC.

This study demonstrates that real-world EHR data can effectively identify Long COVID patients using machine learning algorithms. Our findings underscore the value of integrating heterogeneous EHR sources to support decentralized research efforts. Future research will focus on identifying Long COVID sub-phenotypes—particularly cardiac, respiratory, sleep, and neuropsychiatric presentations—and evaluating the performance of age- and sex-specific models. Additionally, we aim to validate the computable phenotype algorithm in larger real-world datasets to further enhance its clinical and research utility.

## Supplementary Material

ocag115_Supplementary_Data

## Data Availability

Data from the RECOVER Observational Cohorts used in this analysis is available to qualified researchers on the NHLBI BioDataCatalyst platform (see https://recovercovid.org/data). Analysis code is available upon reasonable request to the corresponding author. An open-source R package, longcovidcp (https://github.com/vcastro/longcovidcp), includes functions to compute RECOVER CP scores.
