# Supplementary material for: Characterization and validation of EHR computable phenotypes for Long COVID using patient-reported symptoms: insights from the nationwide RECOVER program
Source: J Am Med Inform Assoc. 2026 Jul 24;33(9):1759–69. doi: 10.1093/jamia/ocag115 (PMC13580738; doi:10.1093/jamia/ocag115)
Supplement: Supplementary file 1 [file ocag115_supplementary_data.docx]

**Title:** Characterization and Validation of EHR Computable Phenotypes for Long COVID Using Patient-Reported Symptoms: Insights from the Nationwide RECOVER Program

**SUPPLEMENTAL MATERIALS**

**Table S1**: Sociodemographic characteristics of RECOVER-Adult participants contributing EHR data compared to participants who did not contribute.

| **Characteristic** | **Contributing EHR Data**  N = 1,501 | **Not Contributing EHR data**  N = 12,673 | **Difference**  **(95% CI)***^1,2^* |
| --- | --- | --- | --- |
| Female sex at birth, n (%) | 1,082 (72) | 9,133 (72) | -0.22% (-2.7% to 2.2%) |
| Age at enrollment, y, Median (IQR) | 46 (36 – 59) | 45 (34 – 59) | 1.1 (0.36 to 1.9) |
| Age category at enrollment, y, n (%) |  |  | 0.04 (-0.02 to 0.09) |
| 18-45 | 740 (49) | 6,419 (51) |  |
| 46-65 | 540 (36) | 4,529 (36) |  |
| >65 | 221 (15) | 1,723 (14) |  |
| Race/ethnicity, n (%) |  |  | 0.42 (0.37 to 0.48) |
| Hispanic | 162 (11) | 2,271 (18) |  |
| Mixed race/Other/Missing | 74 (4.9) | 701 (5.5) |  |
| Non-Hispanic Asian | 76 (5.1) | 742 (5.9) |  |
| Non-Hispanic Black | 90 (6.0) | 1,955 (15) |  |
| Non-Hispanic White | 1,099 (73) | 7,004 (55) |  |
| Prevalent SARS-CoV-2 strain at index, n (%) |  |  | 0.00 (-0.05 to 0.06) |
| Omicron | 1,029 (69) | 8,660 (68) |  |
| pre-Omicron | 472 (31) | 4,013 (32) |  |
| Education level, n (%) |  |  | 0.31 (0.25 to 0.36) |
| Did not complete high school/no diploma | 5 (0.3) | 462 (3.7) |  |
| High school/GED/some college/vocational/technical | 374 (25) | 4,068 (32) |  |
| Bachelor’s/advanced degree | 1,116 (75) | 8,008 (64) |  |
| Rural participant, n (%) | 85 (5.7) | 539 (4.3) | 1.4% (0.15% to 2.7%) |
| Medically underserved area, n (%) | 339 (23) | 3,339 (26) | -3.8% (-6.0% to -1.5%) |
| Long COVID status, n (%) |  |  | 0.21 (0.16 to 0.27) |
| Highly symptomatic Long COVID | 376 (25) | 2,077 (16) |  |
| Some or no Long COVID symptoms | 1,125 (75) | 10,596 (84) |  |

*^1^*2-sample test for equality of proportions with continuity correction; Welch Two Sample t-test; Standardized Mean Difference

*^2^*CI = Confidence Interval

**Figure S1:** Data density distribution by time for all RECOVER EHR data and prospective cohort data. Top 4 panels are EHR data domains and bottom panel is prospective data collected in REDCap using participant surveys and site study staff documentation. Retrospective EHR domains include data from 1972 including historical diagnosis and medication prescriptions.

**Figure S2.** Association of EHR Features with Long COVID status from patient-reported symptoms (LCRI>=11). The y-axis is the negative log of the p-value of the coefficient of the Long COVID status in a logistic regression adjusted for age and sex at birth. (A) Diagnoses PheCodes. (B) RxNorm medication ingredients and (C) Labs and vital signs with flags (N: normal, L: low, H: high).

**A. EHR Diagnosis Features**

**B. EHR Medication Features**

**C. EHR Labs and Vitals Features**

**Figure S3**. Feature coefficients of the LCRI ≥ 11 (highly symptomatic Long COVID) computable phenotype LASSO model.

**Table S3.** Threshold performance metrics in a held-out test set for the Highly symptomatic Long COVID LASSO model. Metrics are shown for a model prediction threshold of 0.44 corresponding to a 95% specificity in the training set.

| **Subgroup** | **Value** | **Spec** | **95% CI LL** | **95% CI UL** | **Sens** | **95% CI LL** | **95% CI UL** | **PPV** | **95% CI LL** | **95% CI UL** | **NPV** | **95% CI LL** | **95% CI UL** |
| --- | --- | --- | --- | --- | --- | --- | --- | --- | --- | --- | --- | --- | --- |
| Overall | All participants in test set | 0.91 | 0.88 | 0.95 | 0.47 | 0.37 | 0.58 | 0.65 | 0.53 | 0.76 | 0.84 | 0.79 | 0.88 |
| Sex at birth | Male | 0.89 | 0.83 | 0.95 | 0.38 | 0.14 | 0.63 | 0.37 | 0.13 | 0.65 | 0.89 | 0.83 | 0.95 |
|  | Female | 0.93 | 0.89 | 0.96 | 0.49 | 0.38 | 0.60 | 0.73 | 0.61 | 0.85 | 0.81 | 0.76 | 0.86 |
| Age at enrollment | >65 | 0.86 | 0.76 | 0.94 | 0.18 | 0.00 | 0.44 | 0.21 | 0.00 | 0.50 | 0.84 | 0.74 | 0.93 |
|  | 46-65 | 0.89 | 0.82 | 0.95 | 0.52 | 0.33 | 0.71 | 0.58 | 0.38 | 0.77 | 0.86 | 0.79 | 0.93 |
|  | 18-45 | 0.96 | 0.91 | 0.99 | 0.50 | 0.37 | 0.62 | 0.82 | 0.68 | 0.94 | 0.82 | 0.76 | 0.88 |
| Medically underserved | No | 0.92 | 0.88 | 0.95 | 0.47 | 0.35 | 0.58 | 0.63 | 0.49 | 0.76 | 0.85 | 0.80 | 0.89 |
|  | Yes | 0.91 | 0.81 | 0.98 | 0.46 | 0.25 | 0.65 | 0.71 | 0.44 | 0.93 | 0.77 | 0.65 | 0.88 |
| Prevalent SARS-CoV-2 variant at index infection | Omicron | 0.93 | 0.89 | 0.96 | 0.44 | 0.27 | 0.62 | 0.47 | 0.29 | 0.64 | 0.92 | 0.88 | 0.95 |
|  | pre-Omicron | 0.88 | 0.80 | 0.95 | 0.48 | 0.36 | 0.60 | 0.79 | 0.65 | 0.90 | 0.65 | 0.54 | 0.74 |
| Race/ethnicity | Non-Hispanic White | 0.90 | 0.86 | 0.94 | 0.46 | 0.34 | 0.58 | 0.60 | 0.47 | 0.73 | 0.84 | 0.79 | 0.89 |
|  | Hispanic | 0.88 | 0.77 | 0.97 | 0.62 | 0.33 | 0.90 | 0.67 | 0.37 | 0.92 | 0.86 | 0.74 | 0.97 |
|  | Non-Hispanic Black | 1.00 | 1.00 | 1.00 | 0.67 | 0.00 | 1.00 | 1.00 | 1.00 | 1.00 | 0.94 | 0.81 | 1.00 |
|  | Non-Hispanic Asian | 1.00 | 1.00 | 1.00 | 0.25 | 0.00 | 1.00 | 1.00 | 1.00 | 1.00 | 0.78 | 0.54 | 1.00 |
|  | Mixed race/Other/Missing | 1.00 | 1.00 | 1.00 | 0.37 | 0.00 | 0.73 | 1.00 | 1.00 | 1.00 | 0.70 | 0.47 | 0.89 |

**Table S4.**  Top EHR Features associated with a Long COVID LCRI symptom (LCRI ≥ 1). The top 20 features for each EHR data domain (diagnosis, procedures, medications, lab tests and vital signs) are shown. Features are ranked by lowest p-values for models adjusted for age and sex at birth.

| **EHR Feature** | **OR** | **SE** | **p-value** | **LCRI** ≥ **1 N=1,317** | **LCRI = 0 N=560** |
| --- | --- | --- | --- | --- | --- |
| **Diagnosis** | | | | | |
| PheCode 798 - Malaise and fatigue | 3.30 | 0.12 | 6.02 × 10^-22 | 436 | 118 |
| PheCode 512 - Other symptoms of respiratory | 2.98 | 0.11 | 9.92 × 10^-22 | 536 | 178 |
| PheCode 512.7 - Shortness of breath | 3.79 | 0.14 | 9.47 × 10^-21 | 342 | 75 |
| PheCode 327.3 - Sleep apnea | 2.93 | 0.14 | 2.28 × 10^-14 | 297 | 89 |
| PheCode 327.32 - Obstructive sleep apnea | 2.82 | 0.15 | 2.35 × 10^-12 | 258 | 77 |
| PheCode 427 - Cardiac dysrhythmias | 2.46 | 0.13 | 2.59 × 10^-12 | 340 | 108 |
| PheCode 339 - Other headache syndromes | 2.53 | 0.13 | 5.27 × 10^-12 | 320 | 91 |
| PheCode 512.8 - Cough | 2.34 | 0.13 | 1.39 × 10^-11 | 351 | 116 |
| PheCode 327 - Sleep disorders | 2.29 | 0.12 | 1.77 × 10^-11 | 364 | 132 |
| PheCode 338 - Pain | 2.52 | 0.14 | 1.58 × 10^-10 | 262 | 78 |
| PheCode 512.9 - Other dyspnea | 3.38 | 0.19 | 3.33 × 10^-10 | 168 | 36 |
| PheCode 300 - Anxiety disorders | 2.17 | 0.13 | 1.21 × 10^-9 | 340 | 110 |
| PheCode 136 - Other infectious and parasitic dx | 3.46 | 0.21 | 2.57 × 10^-9 | 153 | 30 |
| PheCode 292 - Neurological disorders | 2.77 | 0.18 | 6.11 × 10^-9 | 188 | 46 |
| PheCode 296 - Mood disorders | 2.08 | 0.13 | 7.75 × 10^-9 | 329 | 112 |
| PheCode 296.2 - Depression | 2.10 | 0.13 | 1.28 × 10^-8 | 308 | 103 |
| PheCode 427.9 - Palpitations | 2.41 | 0.16 | 1.62 × 10^-8 | 219 | 63 |
| PheCode 300.1 - Anxiety disorder | 2.02 | 0.13 | 1.06 × 10^-7 | 301 | 100 |
| PheCode 495 - Asthma | 2.34 | 0.16 | 1.10 × 10^-7 | 208 | 58 |
| PheCode 761 - Cervicalgia | 2.37 | 0.16 | 1.53 × 10^-7 | 193 | 55 |
| **Labs and Vitals** | | | | | |
| CRP (Normal) | 1.61 | 0.11 | 1.23 × 10^-5 | 514 | 242 |
| Ferritin (Normal) | 1.63 | 0.12 | 2.32 × 10^-5 | 381 | 161 |
| HR (High) | 1.63 | 0.12 | 3.70 × 10^-5 | 377 | 153 |
| Troponin I (Normal) | 1.55 | 0.11 | 6.39 × 10^-5 | 459 | 217 |
| DBP (Normal) | 1.95 | 0.17 | 8.90 × 10^-5 | 867 | 475 |
| BMI (High) | 1.51 | 0.11 | 1.61 × 10^-4 | 458 | 210 |
| Calcium (Low) | 1.68 | 0.14 | 1.78 × 10^-4 | 228 | 91 |
| Ferritin (High) | 2.91 | 0.29 | 2.69 × 10^-4 | 67 | 15 |
| SBP (High) | 1.75 | 0.16 | 3.44 × 10^-4 | 844 | 465 |
| DBP (High) | 1.63 | 0.14 | 3.94 × 10^-4 | 800 | 433 |
| SBP (Normal) | 1.74 | 0.16 | 4.60 × 10^-4 | 847 | 463 |
| Alkaline phosphatase (Normal) | 1.62 | 0.14 | 6.35 × 10^-4 | 808 | 442 |
| Potassium (Low) | 1.64 | 0.15 | 1.06 × 10^-3 | 190 | 73 |
| Creatinine (High) | 1.68 | 0.16 | 1.08 × 10^-3 | 160 | 69 |
| CRP (High) | 1.44 | 0.12 | 1.77 × 10^-3 | 346 | 158 |
| DBP (Low) | 1.40 | 0.11 | 2.17 × 10^-3 | 586 | 298 |
| Cholesterol in Serum (High) | 1.41 | 0.11 | 2.22 × 10^-3 | 436 | 225 |
| Urea nitrogen (Normal) | 1.55 | 0.15 | 2.48 × 10^-3 | 818 | 453 |
| Bilirubin (Normal) | 1.50 | 0.15 | 5.20 × 10^-3 | 817 | 455 |
| Sodium (Normal) | 1.52 | 0.15 | 6.74 × 10^-3 | 833 | 467 |
| **Medications** | | | | | |
| RXNORM 435 - albuterol | 2.45 | 0.11 | 3.97 × 10^-15 | 485 | 171 |
| RXNORM 25480 - gabapentin | 2.69 | 0.14 | 9.45 × 10^-13 | 299 | 88 |
| RXNORM 41126 - fluticasone | 2.17 | 0.12 | 1.97 × 10^-11 | 431 | 158 |
| RXNORM 72625 - duloxetine | 3.68 | 0.21 | 6.34 × 10^-10 | 158 | 29 |
| RXNORM 7646 - omeprazole | 2.17 | 0.14 | 1.99 × 10^-8 | 270 | 89 |
| RXNORM 8640 - prednisone | 1.96 | 0.12 | 2.39 × 10^-8 | 359 | 136 |
| RXNORM 18993 - benzonatate | 2.01 | 0.13 | 1.52 × 10^-7 | 281 | 101 |
| RXNORM 19831 - budesonide | 2.69 | 0.20 | 4.10 × 10^-7 | 145 | 36 |
| RXNORM 42347 - bupropion | 2.20 | 0.16 | 4.38 × 10^-7 | 206 | 63 |
| RXNORM 6470 - lorazepam | 2.13 | 0.16 | 1.52 × 10^-6 | 196 | 63 |
| RXNORM 6227 - lanolin | 0.29 | 0.26 | 1.56 × 10^-6 | 28 | 40 |
| RXNORM 11248 - vitamin B12 | 2.57 | 0.20 | 1.68 × 10^-6 | 137 | 36 |
| RXNORM 704 - amitriptyline | 3.50 | 0.26 | 1.97 × 10^-6 | 99 | 18 |
| RXNORM 18603 - azelastine | 2.43 | 0.19 | 2.71 × 10^-6 | 143 | 40 |
| RXNORM 57258 - tizanidine | 2.91 | 0.23 | 5.43 × 10^-6 | 105 | 24 |
| RXNORM 187832 - pregabalin | 3.54 | 0.28 | 6.35 × 10^-6 | 84 | 16 |
| RXNORM 25255 - formoterol | 2.93 | 0.24 | 6.42 × 10^-6 | 105 | 23 |
| RXNORM 6918 - metoprolol | 2.08 | 0.17 | 1.04 × 10^-5 | 169 | 59 |
| RXNORM 7531 - nortriptyline | 4.83 | 0.36 | 1.15 × 10^-5 | 71 | 9 |
| RXNORM 7213 - ipratropium | 2.19 | 0.18 | 1.21 × 10^-5 | 150 | 46 |
| **Procedures** | | | | | |
| CCS 38 - Other diagnostic procedures | 2.23 | 0.18 | 1.18 × 10^-5 | 145 | 44 |
| CCS.203 - Electrographic cardiac monitor | 2.61 | 0.28 | 5.39 × 10^-4 | 70 | 17 |
| CCS 193 - Echocardiography | 1.70 | 0.15 | 5.61 × 10^-4 | 180 | 73 |
| CCS 220 - Ophthalmologic and otologic proc | 0.68 | 0.14 | 6.52 × 10^-3 | 133 | 115 |
| CCS 199 - Electroencephalogram (EEG) | 3.39 | 0.45 | 6.89 × 10^-3 | 32 | 6 |
| CCS 84 - Cholecystectomy and common | 5.23 | 0.61 | 7.08 × 10^-3 | 26 | 3 |
| CCS 202 - Electrocardiogram | 1.46 | 0.15 | 1.20 × 10^-2 | 171 | 81 |
| CCS 130 - Other diagnostic procedure | 2.04 | 0.32 | 2.54 × 10^-2 | 48 | 13 |
| CCS.70 - Upper gastrointestinal endoscopy | 1.64 | 0.23 | 3.09 × 10^-2 | 75 | 29 |
| CCS 134 - Cesarean section | 0.41 | 0.43 | 3.58 × 10^-2 | 11 | 12 |
| CCS 139 - Fetal monitoring | 0.35 | 0.51 | 4.05 × 10^-2 | 7 | 9 |
| CCS 7 - Other diagnostic nervous | 1.46 | 0.19 | 4.70 × 10^-2 | 103 | 45 |
| CCS 183 - Routine chest x-ray | 1.57 | 0.24 | 5.50 × 10^-2 | 66 | 28 |
| CCS 208 - Radioisotope pulmonary scan | 2.16 | 0.41 | 6.16 × 10^-2 | 26 | 8 |
| CCS.47 - Diagnostic cardiac catheterization | 2.41 | 0.47 | 6.39 × 10^-2 | 20 | 6 |
| CCS 20 - Other intraocular therapy | 2.31 | 0.47 | 7.55 × 10^-2 | 20 | 6 |
| CCS 30 - Tonsillectomy and adenoidectomy | 2.98 | 0.63 | 8.51 × 10^-2 | 16 | 3 |
| CCS 80 - Appendectomy | 2.72 | 0.64 | 1.16 × 10^-1 | 15 | 3 |
| CCS 6 - Decompression peripheral nerve | 2.71 | 0.65 | 1.25 × 10^-1 | 12 | 3 |
| CCS 198 - MRI | 1.36 | 0.20 | 1.26 × 10^-1 | 85 | 42 |

**Figure S4.** Secondary LCRI ≥ 1 phenotype model performance. (A) Receiver operating characteristic plot of ICD-10-CM U09.9 Post COVID-19 Conditions code (red) and the machine learning computable phenotyping model (blue). (B) Calibration plot of the machine learning model.

**Figure S5**. Feature coefficients of the LCRI ≥ 1 secondary computable phenotype LASSO model.
